# A theoretical insight to understand the molecular mechanism of dual target ligand CTA-018 in the chronic kidney disease pathogenesis

**DOI:** 10.1371/journal.pone.0203194

**Published:** 2018-10-04

**Authors:** Selvaraman Nagamani, Karthikeyan Muthusamy

**Affiliations:** Department of Bioinformatics, Alagappa University, Karaikudi, India; University of Louisville, UNITED STATES

## Abstract

The level of the vitamin D in the bloodstream is regulated by cytochrome P450 enzyme 24-hydroxylase A1 (CYP24A1). Over expression of CYP24A1 enzyme is correlated with vitamin D deficiency and resistance to vitamin D therapy. Chronic kidney disease (CKD) patients are commonly reported with the above said expression variations. This deregulation could be solved by ligands that act as a vitamin D receptor (VDR) agonists and CYP24A1 antagonists. Posner et al., (2010) first time reported two new vitamin D analogues namely CTA-091 and CTA-018 to inhibit CYP24A1. The CTA-018 inhibited CYP24A1 with an IC_50_ 27 ± 6 nM (10 times more potent than the ketoconazole (253 ± 20 nM)). CTA-018 induced VDR expression (15-fold lower than 1α,25(OH)_2_D_3_) and is under phase II clinical trial, whereas CTA-091 was not able to efficiently induce the VDR expression (>2000 nM). To explore the molecular mechanism, binding specificity of these two vitamin D analogues along with native ligand was extensively studied through *in silico* approaches. Through molecular dynamics simulations studies, we shown that the sulfonic group (O = S = O) in the side chain of CTA-018 plays an important role in the regulation of VDR agonistic activity. The electron lone pairs of the sulfonic group that interacted with His393 lead to be a factor for agonistic mechanism of VDR activity. Compared to azol-based compounds, CTA-018 binds the different sites in the CYP24A1 binding cavity and thus it could be a potent antagonistic for CYP24A1enzyme.

## Introduction

Worldwide, Chronic kidney disease (CKD) is a major public health problem; it is one of the high-risk factors for hypertension and diabetes [1] patients. Progressive reduction of circulating 1α,25-dihydroxy vitamin D_3_ (1α, 25(OH)_2_D_3_) and 25-hydroxyvitamin D_3_ (25(OH)_2_D_3_) are common expression variation observed in CKD patients [2]. Several vitamin D analogues like 1α,25(OH)_2_D_3_ (i.e., calcitriol) and 25(OH)_2_D_3_ (i.e., cholecalciferol) were used for the treatment of secondary hyperparathyroidism (sHPT) among CKD patients but their efficacy is often limited since it triggers hypercalcemia [3] and also the efficacy of these drugs is largely determined by efficient binding with VDR.

The VDR transcriptional activity has been regulated by numerous factors such as ligand binding affinity, ligand-dependent recruitment of co-activators or dissociation of repressors, efficiency of the ligand uptake into the target cell, tissue specificity and different metabolism of ligands [4]. The efficacy of VDR therapies is also regulated by the intracellular factors like Extra-renal 1α-hydroxylase (CYP27B1) it permits localized synthesis of additional 1α,25(OH)_2_D_3_; Cytochrome P450 enzyme 24-hydroxylase (CYP24A1) metabolizes 1α,25(OH)_2_D_3_, 25(OH)D_3_ and administers analogues by hydroxylation reaction [5].

The CKD pathogenesis has influenced by the genes FGF23, CYP24A1 and VDR [2,6]. FGF23 is the recently reported regulator of phosphate and mineral metabolism; it mainly regulates the renal phosphate excretion. FGF23 levels are increased among CKD patients and many cross sectional studies demonstrated that an inverse relationship have observed in glomerular filtration rate (GFR) with an inverse kidney function [7,8]. The increased level of FGF23 leads to the over expression of CYP24A1 mRNA in the kidney [9,10]. The of 25 hydroxyvitamin D_3_ (25–OHD_3_) and its hormonal form, 1, 25- di hydroxyvitamin D_3_ (1,25-(OH)_2_D_3_) was catabolished into 24-hydroxylated products for excretion by the enzyme CYP24A1[2]. Further, the active form of the VDR mediates a wide variety of biological actions such as cell proliferation and differentiation, calcium homeostasis, immune modulation, neurological functions and bone mineralization [11]. The over-expression of the CYP24A1 leads to dysfunction of the VDR as it over metabolized the 25OHD_3_ and 1,25(OH)_2_D_3_. Thus, CKD patients ought to experience vitamin D deficiency and subsequent osteoporosis [12].

**Fig 1:**
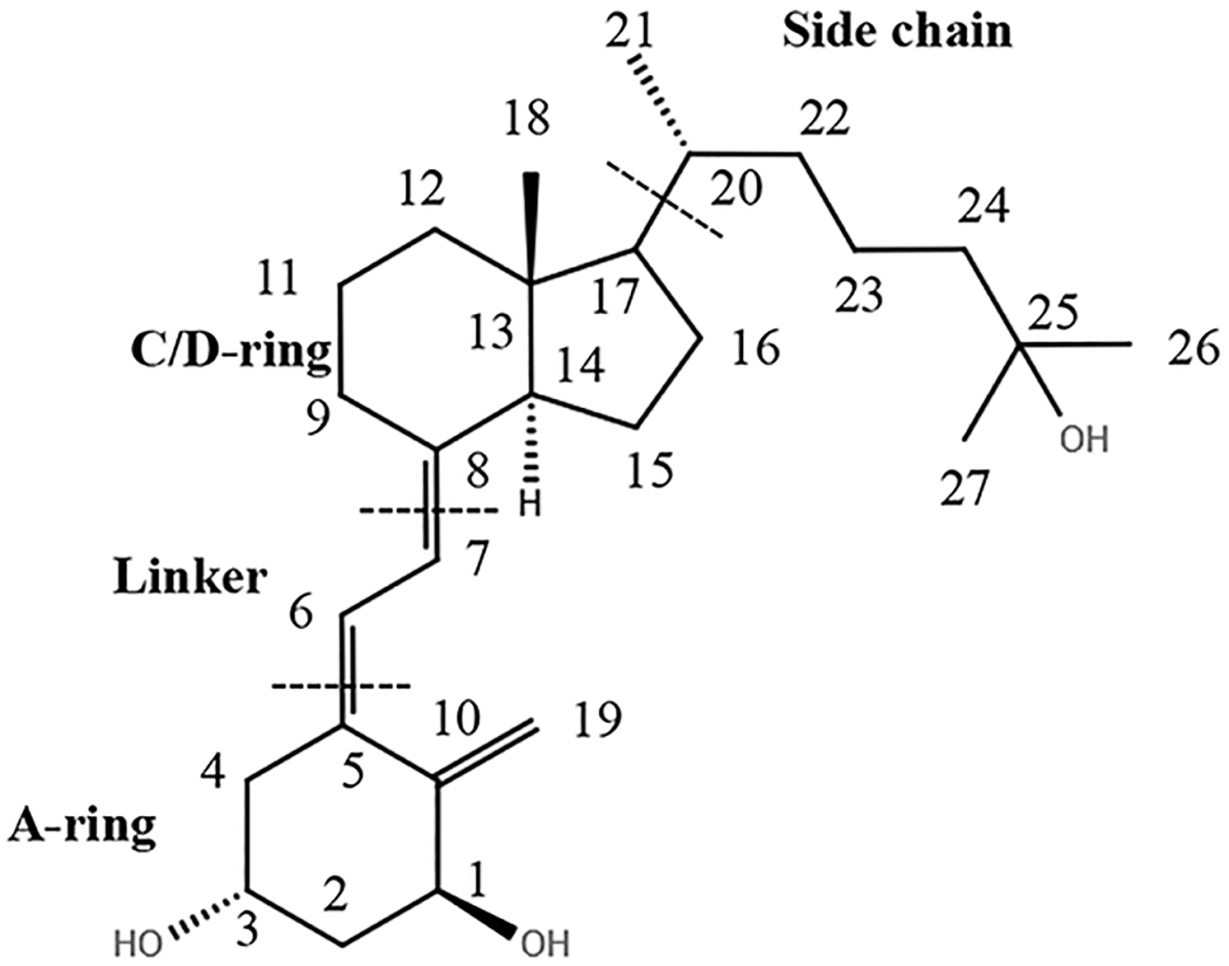
The basic skeleton of vitamin D analogues.

A 25-methyl ether version of the natural hormone 1α,25(OH)_2_D_3_ was first reported by the DeLuca group in 1987 which retained most of the pro-differentiation activities of 1α,25(OH)_2_D_3_ [13]. Till date, numerous vitamin D analogues are reported to show agonistic activity against the VDR. The common structure of Vitamin D analogues comprises four major classes: The A ring analogues [14], the seco B ring analogues [15], the C/D ring analogues [16], and the side chain analogues [17]. Most of the analogues have been modified around the side chain, since it determines the agonistic and antagonistic nature of the compounds. The basic architecture of vitamin D analogues is shown in Fig 1.

The three-dimensional structure of the VDR was modeled by Yamamoto, et al. [18] and the importance of His397 interaction in the agonistic mechanism was identified by site directed mutagenesis. Darimont, et al. (1998) [19] reported that helix 12 (Leu325 –Ser401) folding plays a crucial role and ligand modulated interface for interaction with the co-activator proteins. Some other computational studies also had shown the good VDR agonistic activity of vitamin D analogues [20]. Cumulatively all these studies indicate that side chain modification might have vital role in VDR activity.

Posner, et al. [21] had designed two promising vitamin D analogues: The sulfoximine MK-24(S)-S(O)(NH)-Ph-1 (CTA091—potent, selective and non-calcemic inhibitor) and the sulfone GHP-GH-16,23-diene-25S02-1 (CTA018/MT2832—potent activator of VDR-mediated transcription and low calcemic inhibitor of CYP24A1) CTA-018, which has a high affinity with VDR (15-fold lower than 1α25(OH)_2_D_3_) as well as CYP24A1 (IC_50_ 27 ± 6 nM, about 10 times more potent than the non-selective CYP24A1 inhibitor ketoconazole (253 ± 20 nM). The CTA-091 did not show any measurable affinity for the VDR whereas CTA-018 bound to the VDR with 15-fold lower affinity than 1α,25(OH)_2_D_3_. The *in vitro* and *in vivo* studies clearly explained that both the vitamin D analogues are readily bound to the substrate binding pocket of CYP24A1 and target the HEM group at the catalytic core of the enzyme. However, CTA-018 induced VDR-mediated gene expression rather than CTA-091, as it is unable to induce the VDR expression. Consistent with these findings, CTA-018 is a selective drug candidate to treat CKD and sHPT among people who regularly undergo hemodialysis.

The ligand binding sites (His305 (His301 in rat) and His397 (His393 in rat)) of the VDR was identified by Kakuda, et al. [22] using X-ray crystallographic studies. VDR activity is mainly governed by these two amino acids. The biological and biochemical properties of CTA-018 was studied in detail but dynamic behavior in the active site of VDR and CYP24A1 remains unexplored.

In this work, specific role of the CTA-091 and CTA-018 compounds with the VDR and CYP24A1 proteins were studied using molecular docking, molecular dynamics simulations, binding free energy calculations, and density functional theory (DFT) calculations. These two vitamin D analogues along with the reference ligands (we consider 1α25(OH)_2_D_3_ as a reference ligand for VDR and ketoconazole as a reference ligand for CYP24A1) were docked in the active site of VDR and CYP24A1. Further, 200 ns MD simulations were carried out on eight different models (VDR/Apo form, VDR/1α25(OH)_2_D_3_, VDR/CTA-091, VDR/CTA-018, CYP24A1/Apo form, CYP24A1/Ketoconazole, CYP24A1/CTA-091, and CYP24A1/CTA-018) in order to assess the structural and dynamical changes in the active sites of both the protein complexes. A total of 100 different molecular trajectories were collected from the molecular dynamics study. A molecular mechanics-generalized born/surface area (MM-GBSA) analysis was carried out on the basis of the collected MD trajectories for six different models. Finally, DFT calculations were carried out to analyze the activity of the molecules. The outcome of this paper will pave the way to understand the behavior of newly identified compounds (CTA-091 & CTA-018) at the atomic level.

## Materials and methods

All computational analyses were carried out on the red hat 5.1 Linux platform.

### Preparation of protein structure

The rat CYP24A1 was selected for study because human CYP24A1 protein structure is not available and the newly identified vitamin D analogues have tested against rat animal model. The crystal coordinates of rat VDR in complex with partial agonist 26-adamantyl-23-yne-19-norvitamin D were downloaded from the Protein Data Bank. The sequence identity of human and rat VDR show 94% similarity in its sequence and the active site region is similar to both the species. For easy reference, we selected both the proteins from rat species. Thus, we performed our *in silico* analysis on rat crystal structures. The 3D structures of the vitamin D receptor (PDB id –3A2H) [22] and CYP24A1 (PDB id– 3K9V) [23] were retrieved from the Protein Data Bank and further prepared by protein preparation wizard using OPLS_2005 force field [24]. The VDR crystal structure contains numerous missing residues. Thus, while preparing, the missing residues were modeled using PRIME module of Schrodinger [25]. In CYP24A1 protein, the localized charge on the Iron was chosen as Fe^2+^. Initially, chemical accuracy was ensured for the structure. Further, hydrogen atoms were added and unwanted water molecules beyond 5Å were removed from the protein structure. Further, side chains that are neither close to the binding cavity nor involved in the formation of salt bridges were neutralized in order to reduce the CPU time. The energy was minimized until the average root mean square deviation (RMSD) of the non-hydrogen atoms reached 0.3 Å. The minimized structure should not deviate more from the original crystal structure. Thus, we kept 0.3 Å as the cut-off value for the energy minimization.

### Ligand structure prediction

**Fig 2:**
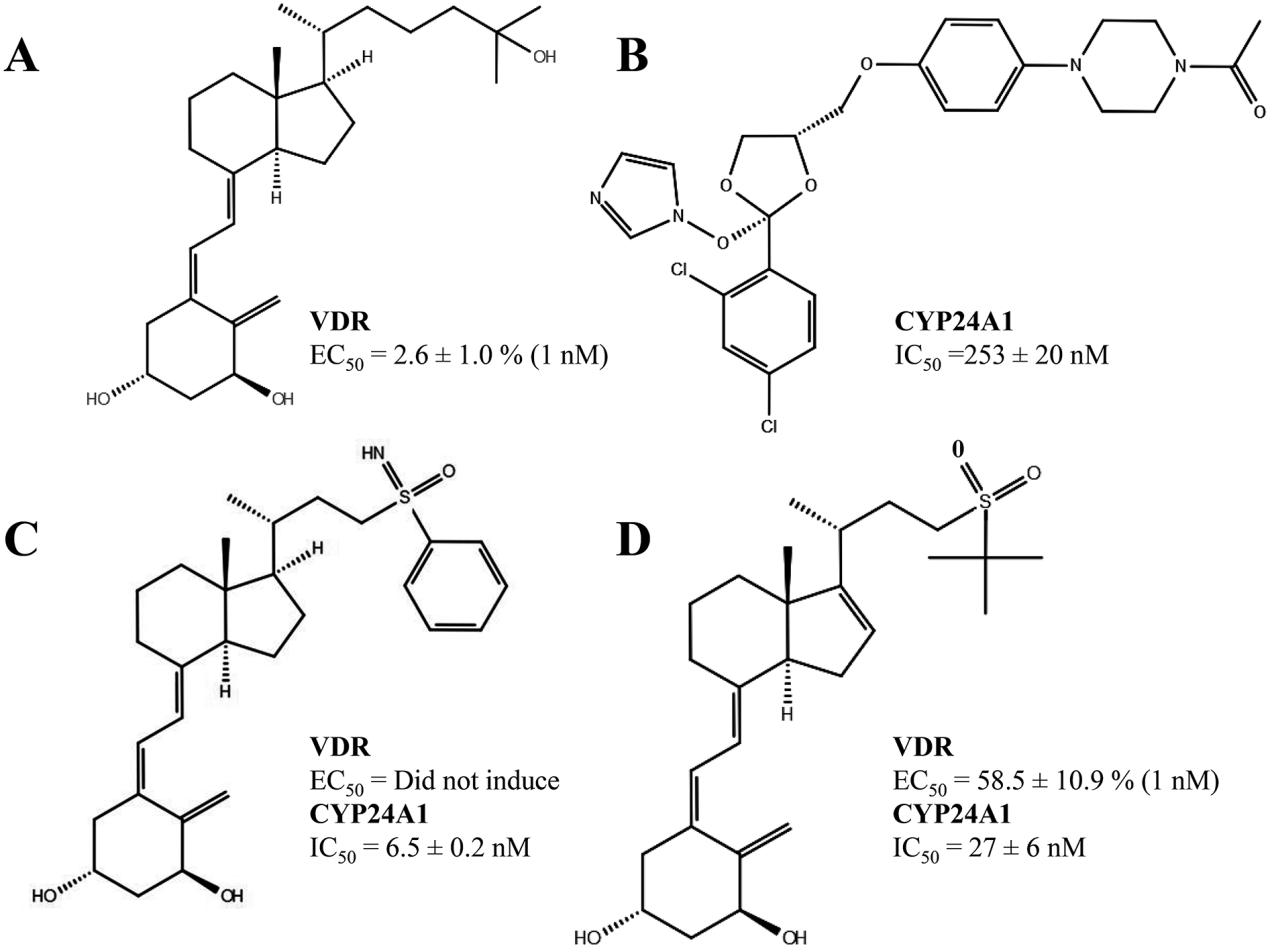
The 2D diagram of the drug molecules (A) 1α25(OH)_2_D_3,_(B) ketoconazole,(C) CTA-091 and (D) CTA-018 used in this study and their corresponding EC_50_ against VDR and IC_50_ against CYP24A1.

Active vitamin D (CID—5280453) CTA-091, CTA-018 (CID 10672195), ketoconazole (CID– 456201) compounds were prepared using Ligprep 2.8 [26] module in Schrodinger maestro. Hydrogen atoms were added to the ligand molecules and the bond order of these ligands was fixed by LigPrep module. Epik was used to ionize the ligands with the pH range of 5.0–9.0. The individual stereotypes of each ligand were extensively analyzed by generating the most probable tautomers and possible stereo isomers [26, 27]. The 2D diagrams of the four compounds are shown in Fig 2.

### Molecular docking simulations

Two different sets of docking studies were performed, In the first set, 1α25 (OH)_2_ D_3_ (reference ligand), CTA-091, and CTA-018 were docked in the VDR active site. In the second set, ketoconozole (reference ligand), CTA-091, and CTA-018 were docked in the CYP24A1 active site. The van der Waals radii of receptor atoms were scaled by 1.00 Å with a partial atomic charge of 0.25 Å. Grid boxes were generated at the centroid of ligand present in VDR and CYP24A1 active sites. All the docking protocols were carried out with the extra precision (XP) mode of Glide docking which docked ligand flexibly. We scaled van der Waal radii of receptor atoms by 1.00 Å with a partial atomic charge of 0.25 Å. A grid box with coordinates X = 11.44 Å, Y = 10.95 Å, Z = 7.42 Å (box range 28.30 Å) was generated at the centroid of the active site in case of VDR and X = 12.52 Å, Y = 11.26 Å, Z = 9.87 Å (box range 30.25 Å) in case of CYP24A1 models. Extra precision (XP) mode of the flexible docking was used for all docking protocol. The Glide XP docking methodology was adopted as like explained in Singh and Muthusamy [28]. The docked complex with lowest energy was selected for further studies.

### Molecular dynamics simulations

Eight models (VDR_apo_, VDR_1α,25(OH)2D3_, VDR_CTA-091_, VDR_CTA-018,_ CYP24A1_apo,_ CYP24A1_ketoconozole_, CYP24A1_CTA-091_ and CYP24A1_CTA-018_) were given as input structure for molecular dynamics (MD) simulation studies. All MD simulations were carried out in GROMACS 4.6.3 [29] with the united atom Gromos9643a1 force field in NVT and NPT environment under the periodic boundary conditions. The ligand coordinates file and ligand topology file were generated externally in the PRODRG server and then it was included with the GROMACS topology file. Each of the eight models was placed in a cubic box containing pre-equilibrated water molecules described by the simple point charge water model. The cubic box dimension is 10.60 × 11.21 × 10.51 (all in Å) in the case of VDR models and 11.24 × 12.54 × 10.58 (all in Å) in the case of CYP24A1 models. In the VDR, three Na^+^ ions were replaced with water molecules, whereas in the CYP24A1 enzyme five Cl^-^ ions were replaced with water molecules at random positions in order to make the model system neutral. The leap-frog integrator was used for MD simulation step. The whole system was minimized on the basis of the steepest descent method with 50,000 minimization steps and a tolerance of 1000 kJ mol^-1^ nm^-1.^ Particle mesh Ewald (PME) method was applied for the electrostatic and van der Waals (vdW) interactions with a cutoff distance of 1.0 nm for short-range neighbor list (rlsit) and 1.0 nm for coulomb cutoff (rcoulomb) and 1.0 nm for the vdW interactions. The Nose-Hoover heat bath with a tau value of 0.5 ps and Parrinello-Rahman barostat with a tau value of 1.0 ps were used for the temperature and pressure coupling respectively. In the CYP24A1 enzyme, HEM group was parameterized with the default parameters available in Gromos9643a1 force field.

A total of 200 ns simulations were carried out in the MD production step. Constant pressure and temperature (298K) was maintained for all MD simulations. The 200 ns MD simulation trajectories were saved at 2 fs time interval throughout the whole simulation period and it was analyzed for further calculations. Secondary structure was analyzed using DSSP [30]. Figures of the molecular structures were generated with Pymol [31] and Schrodinger Maestro [32].

### Essential dynamics

The dominant motion of a protein over the GROMACS simulation was determined using the essential dynamics technique based on the statistical principal component analysis (PCA) [33]. A covariance matrix was constructed from the atomic fluctuations in a trajectory, which yielded a set of eigenvectors and eigenvalues. The translational and rotational motions of the trajectory were removed before proceeding with this step. The eigenvectors indicate the directions of motions and the eigenvalues indicate the amount of motion along with the eigenvectors. Most of the variance in the first and second principal components is typically explained by PCA analysis. The time dependence of protein motion along with eigenvectors was explained by projections of the trajectories and individual eigenvectors [34].

### Free energy calculations

100 different conformers were generated for each model from MD trajectories and binding free energy was calculated for each conformer. Prime MM/GBSA method was used to calculate the binding free energy for a set of ligands to receptor. The energy was minimized by using the local optimization feature in Prime, and the energies of the complex were calculated using the OPLS_2005 force field and the GB/SA continuum solvent model. The binding free energy is calculated as follows [35,36],

$$\Delta G_{bind}=\Delta_{E}+\Delta G_{solv}+\Delta G_{SA}$$ (1)

$$\Delta E=E_{complex}-E_{protein}-E_{ligand}$$ (2)

where E_complex_, E_protein_ and E_ligand_ are the minimized energies of the protein-ligand complex, protein and ligand respectively. The methodologies were adopted as explained in the publications [28,37]. The average mean binding free energy was considered for each complex.

### Density Functional Theory calculations

The four compounds were subjected to the Density Functional Theory (DFT) calculations. DFT calculations can be used to calculate the electronic molecular features such as molecular electrostatic map, electron density, and frontier molecular orbital density fields (i.e., HOMO, LUMO), which can explain the molecular properties and biological activity. The DFT calculations were performed with Jaguar [38] on the basis of solvation state. The DFT was analyzed through Becke’s three-parameter exchange potential and the Lee-Yang-Parr correlation functional (B3LYP) using basic set of 6-31G**++ level (Jaguar) in PBF solvation. In the present work, 3D-molecular electrostatic potentials (MESP) V(r) at a point r because of a molecular system with nuclear charges {ZA} located at {RA} and the electron density ρ (r) were derived using the following equation.

$$V(r)=\sum_{A=1}^{N}\frac{Z_{A}}{\left|r-R_{A}\right|}-\int \frac{\rho (r\prime )d^{3}r\prime }{\left|r-r\prime \right|}$$ (3)

In this equation, N represents the total number of nuclei in the molecule and the two terms refer to the bare nuclear potential and the electronic contributions, respectively. We computed the Jaguar dipole moment, Molecular electrostatic properties, lowest unoccupied molecular orbital (LUMO), including MESP and the highest occupied molecular orbital (HOMO) energy. The electrostatic potentials were calculated by van der Waals contact surface area of the molecule. The overall molecular size and the positive electrostatic potentials are indicated by color coded surface values. The most negative electrostatic potential is colored deep red and the most positive electrostatic potential is colored deep blue. The intermediate yellow, orange, green shades indicate the intermediate ranges of reactivity [38].

## Results and discussion

### Molecular docking and molecular dynamics—Phase I

In the Phase-I, we displayed and discussed the VDR models results.

#### Molecular docking and binding mode analysis of VDR

**Fig 3:**
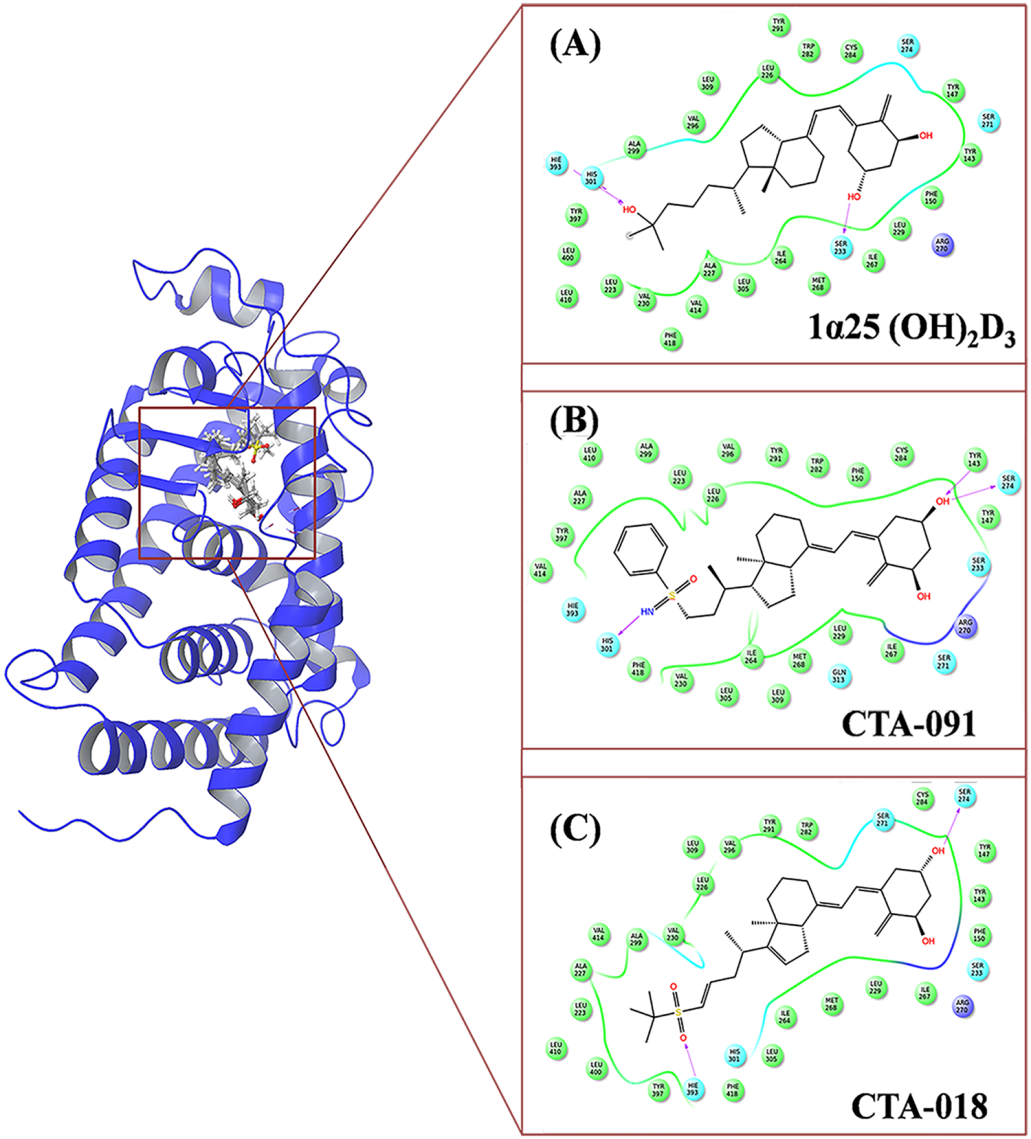
The 2D representation of the compounds (A) 1α25(OH)_2_D_3_, (B) CTA-091 and (C) CTA-018 in the active site of VDR.

The grid was generated for VDR (PDB id– 3A2H) around the co-crystallized ligand (TEI-9647). The 1α, 25 (OH)_2_D_3_, CTA-091, and CTA-018 were docked in the VDR active site. In the VDR active site, the ligands were in good agreement with the available crystal structures. In the VDR_1α25(OH)2D3_ complex, a hydroxyl group of (-OH) polar/uncharged Ser233 interacted with the ligand molecule (0.18 nm, HO^**…**^HO). In addition, nitrogen atom from the cyano group (-CN) of charged His301 interacted with the hydrogen atom of the side chain hydroxyl group (-OH) in the ligand molecule (0.19 Å, CN^**…**^HO). Another hydrogen atom from the amino group (-NH) of charged His393 interacted with the side chain oxygen atom in the hydroxyl group (-OH) of the ligand molecule (0.19 Å, CN^**…**^OH) (Fig 3a). In the case of VDR_CTA-091_, we could find a major interaction with His301. The nitrogen atom from the cyano group (-CN) interacted with the hydrogen atom in the hydroxyl group of ligand molecule side chain (0.19 Å, CN^**…**^HO). The hydrogen atom from the hydroxyl group (-OH) of Tyr143 interacted with the oxygen atom of the ligand molecule (0.22 nm, OH^**…**^OH). One more additional interaction was observed between the oxygen atom from the hydroxyl group of Ser274 and the hydrogen atom of the ligand molecule (0.21 nm, HO^**…**^HO) (Fig 3b). In VDR_CTA-018_, the oxygen atom from the hydroxyl group (-OH) of uncharged Ser274 was interacted with the hydrogen atom in the hydroxyl group (-OH) of the ligand molecule (0.19 nm, HO^**…**^HO). Notably, the hydrogen atom from the amino group (-NH) of charged His393 interacted with an oxygen atom of the sulfonyl group (O = S = O) in the ligand molecule (0.20 nm, NH^**…**^ O = S = O) (Fig 3c). The docking results are shown in Table 1.

**Table 1:** Glide XP docking results with interacting amino acids in the active site of VDR and CYP24A1.

Models	Glide XP docking score	Glide XP energy (kcal/mol)	Glide XP Emodel (kcal/mol)	Interacting amino acids D-H^…^A
VDR _1α,25(OH)2D3_	-15.45	-59.62	-85.36	(His393) NH^**…**^OH OH^**…**^ CN (His301) OH ^**…**^OH (Ser233)
VDR _CTA-091_	-15.44	-59.51	-77.69	NH^**…**^CN (His301) (Tyr143) OH^**…**^OH OH^**…**^HO (Ser274)
VDR _CTA-018_	-13.47	-48.48	-80.54	OH^**…**^HO (Ser274) (His393) OH^**…**^O = S = O
CYP24A1 _ketoconazole_	-5.55	-49.21	-78.66	NH^**…**^O = C (Glu329) π–π (Arg 128)
CYP24A1 _CTA-091_	-5.82	-40.48	-53.30	OH^**…**^O = C (Met246) OH^**…**^O = C (Glu329)
CYP24A1 _CTA-018_	-5.47	-37.64	-47.45	OH^**…**^O = C (Met246)

The experiential results shown that, His393 and His301 are the important amino acids that control the VDR agonistic and antagonistic activities [22]. The folding of helix 12 is facilitated by the H-bond interaction with His393 and it was considered as a key interaction required for folding. This corrected folding is responsible for the VDR agonistic activity. In addition, through mutational analyses, it was found that His301 interaction was essential for the VDR antagonistic activity. Interestingly, this molecular docking result is very well correlated with the results of crystallographic study. In VDR_1α25 (OH)2D3,_ we could find both interactions with His301 and His393, thus, it may act as agonist. However, in VDR_CTA-091,_ the major interaction was only with His301 which made it as a full antagonist. InVDR_CTA-018,_ we found the interaction was only with His393 because of the two oxygen atoms. Thus, the compound CTA-018 is a more potent VDR agonist than 1α25(OH)_2_D_3_ and CTA-091.The interaction diagram of the drug molecules in the active site of VDR is shown in Fig 3. Further, molecular dynamics simulation studies were performed to study the importance of all the particular ligands.

Moreover, the co-crystallized compound (TEI-9647) in the VDR active site is structurally similar with the newly identified compounds (CTA-091 & CTA-018). In order to elucidate the binding site orientation of these compounds in the VDR active site, it was displayed the superposition of the co-crystallized compound with the three different compounds (1α25(OH)_2_D_3_, CTA-091, CTA-018). The binding site orientation is shown in the S1 Fig.

#### Blind docking analysis

Before MD simulations, binding pocket of the VDR model was analyzed as it contains two different binding sites for two specific functions. In the primary site, calcitriol binds and functions as a ligand-regulated transcription factor to stimulate intestinal calcium absorption, bone calcium resorption, *etc*. In the secondary site, the bile acids (lithocholic acid (LCA) and 3-keto-LCA) bind and protect from their harmful effects in the intestine [39]. In order to elucidate the binding specificity of our studied molecule, we performed blind docking without importing active site information. Induced fit docking (IFD) protocol was used, all the three vitamin D analogues (1α25(OH)_2_D_3_, CTA-091, and CTA-018) were bound in the primary site which is essential for the VDR agonistic activity. It confirms that, the compounds CTA-091 and CTA-018 were binding at the primary binding site. The binding mode of the three different compounds in the active site of VDR is depicted in the S2 Fig.

#### Structural flexibility in VDR

**Fig 4:**
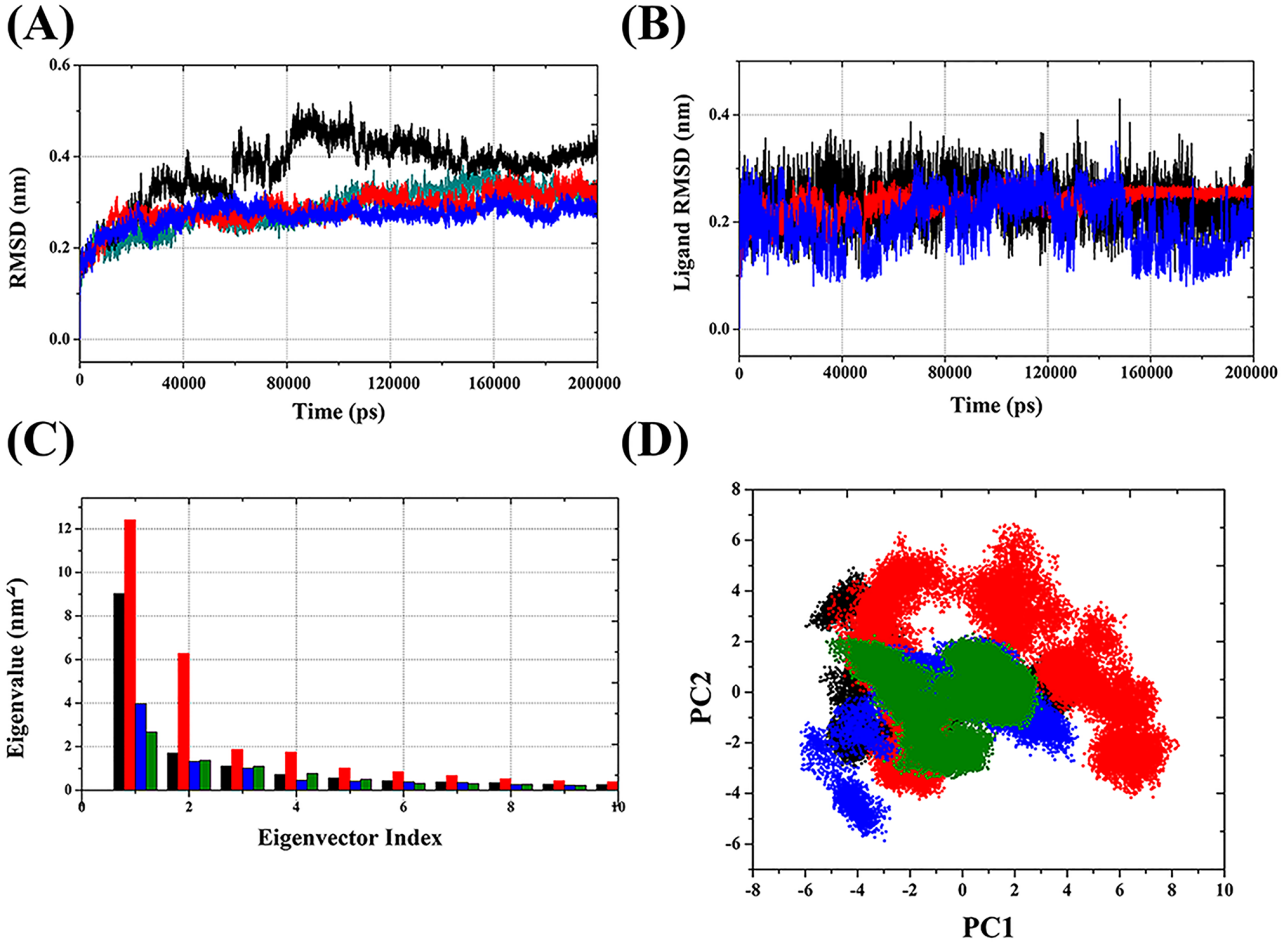
**Time evolution of backbone RMSD (A) and ligand RMSD (B) of VDR models.** VDR_apo_ (cyan), VDR_1α25(OH)2D3_ (black), VDR_CTA-091_ (red), VDR_CTA-018_(blue). Eigenvector index versus Eigenvalue index for the first ten eigenvectors of the VDR model (C) and Projection of the motion of VDR models (D). VDR_apo_ (green), VDR_1α25(OH)2D3_ (black), VDR_CTA-091_ (red), VDRCTA-018(blue).

The degree of conformational drift in the protein was assessed by the backbone atoms with respect to the initial structure as a function of time. The backbone RMSD plots of the four VDR models (VDR_apo_ (cyan), (VDR_1α,25(OH)2D3_ (black), VDR_CTA-091_ (red), and VDR_CTA-018_ (blue)) are shown in Fig 4a. Initially, in the first 5000ps, the RMSD was raised due to “relaxation” of the models in water environment, which is commonly observed in all MD simulation types. The RMSD did not fluctuate convincingly, illustrating an average RMSD of less than 0.5 nm for the overall simulation period.

The largest fluctuation was observed in VDR_1α,25(OH)2D3_ (black). When the RMSD of VDR_1α,25(OH)2D3_ (black) was gradually increased from 40 ns it fluctuated drastically between 80 ns and 120 ns. In contrast, the RMSD of the VDR_CTA-091_ (red) and VDR_CTA-018_ (blue) produced reasonably stable conformation in the overall simulation period. Initially, RMSD oscillated between 0 and 40 ns and showed stable conformer after 40 ns. Although the RMSD of VDR_CTA-018_(blue) fluctuated drastically at 25 ns, after that it maintained stable conformer between 0.20 nm and 0.30 nm. After 40 ns both the newly identified compounds (CTA-091and CTA-018) produced stable conformations as VDR_apo_ (cyan).

#### Conformational changes of ligands in VDR active site

To assess the conformational changes of the ligand molecule in the VDR active site, the RMSD of ligand molecules was calculated with respect to the starting structure as a function of time. The RMSD plots of the three VDR ligand models (1α,25(OH)_2_D_3_(black), CTA-091 (red)_,_ CTA-018 (blue)) are shown in Fig 4b. As mentioned above, His393 and His301 are important amino acids that control the agonistic and antagonistic activity of the VDR action [22]. Using this rationale, we explain the behavior of the three different VDR ligand models. Although all the structures fluctuated in an inappropriate way to each other, they all showed an average RMSD of less than 0.40 nm for the entire simulation period.

The largest fluctuation was observed in VDR_CTA-018_ (blue), which indicates greater flexibility of this compound. The CTA-018 molecule has O = S = O unit which can act as a hydrogen bond acceptor. In due course, the two oxygen atoms of O = S = O were involved in an interaction with hydrogen atoms of His393 and functioned as an agonist for VDR action (Fig 4b). However, not entirely, but alternative formation of hydrogen bonds between the His393 and the two hydrogen bond acceptors (O = S = O) led to more fluctuation in the overall simulation time (0.15 nm to 0.30 nm). In VDR_CTA-091_(red)_,_ the RMSD did not fluctuate significantly and it maintained a proper simulation structure (< 0.30 nm) after some initial fluctuations. The presence of two hydrogen bond acceptors (O = S = O) and one hydrogen bond donor (-NH) of VDR _CTA-091_ (red) formed this stable conformation (Fig 4b). The hydrogen atom from the amino group (-NH) interacted well with the nitrogen atom of His 301, which induced antagonistic activity. The thiol group (O = S = O) did not form any hydrogen bond with the VDR _CTA-091_ (red). Based on this theoretical finding, we reasoned that VDR _CTA-091_ (red) could not induce VDR-mediated gene expression as reported by Posner, et al. [21]. In the VDR_1α,25(OH)2D3_ complex, two hydrogen bonds were observed. However, the structure has a hydrogen bond donor and a hydrogen bond acceptor in the same functional group (-OH). The hydrogen atom from the—OH group interacted with the nitrogen atom of His301 and the oxygen atom from the same group interacted with the nitrogen atom of His393. These interactions made VDR_1α,25(OH)2D3_ (black) as partial agonist and partial antagonist. Due to the presence of His301 and His393 interactions VDR_1α,25(OH)2D3_(black) is more fluctuating than VDR _CTA-091._ Explicit analyses of ligand RMSD in the active site of VDR are shown in Table 2 and in S3 Fig.

**Table 2:** Explicit analyses of ligand RMSD in 200 ns MD simulation (All units are in nm).

Complex	Mean	Standard error	Median	Standard deviation	Minimum	Maximum	Confidence level (95.0%)
VDR _1α,25(OH)2D3_	0.24	0.0001	0.24	0.03	0.000006	0.43	0.24
VDR _CTA-091_	0.24	0.0001	0.24	0.02	0.0004	0.29	0.24
VDR _CTA-018_	0.20	0.0004	0.20	0.05	0.0004	0.35	0.20
CYP24A1 _ketoconazole_	0.19	0.0001	0.19	0.03	0.0004	0.27	0.33
CYP24A1 _CTA-091_	0.18	0.0001	0.19	0.03	0.0004	0.27	0.34
CYP24A1 _CTA-018_	0.31	0.00008	0.31	0.02	0.0004	0.38	0.32

#### Principal component analysis (PCA)

Followed by molecular dynamics simulations, the dimensionality of the coordinate data was reduced to identify the configurational space, which contains only a few degrees of freedom where anharmonic motion occurs. The PCA method takes the trajectory of a molecular dynamics simulation and extracts the dominant modes in the motion of the molecule. These pronounced motions correspond to correlate vibrational modes or collective motions of groups of atoms in normal mode analysis. The overall translational and rotational motions in the MD trajectory are eliminated by a translation to the average geometrical center of the molecule and by least squares fit superimposition “onto” a reference structure. The most important motions of the protein are extracted from the trajectory through PCA of the Cartesian coordinate covariance matrix, yielding eigenvectors and corresponding eigenvalues [34, 40].

The global motions of the three different VDR protein-ligand complexes were compared by the PCA. The results are shown in Fig 4c and 4d respectively. The results exhibit that, the first two eigenvectors (color coding) displayed more than 90% of the collective motions of the backbone protein atoms (Fig 4c). Projections of the MD trajectories obtained at 298K onto the principal components PC1 and PC2 map the motions of our VDR models in phase space: VDR_apo_(black) VDR_1α,25(OH)2D3_ (red), VDR_CTA-091_(blue) and VDR_CTA-018_ (green)_._ A comparison of the data in Fig 4d (~5–6 units), based on the width of the point clusters, shows that (a) VDR_CTA-091_ (blue) and VDR_CTA-018_ (green) exhibit the same type of phase space coverage compared to VDR_1α,25(OH)2D3_ (red). (b) The VDR_CTA-091_ (blue) and VDR_CTA-018_ (green) complexes form similar phase space coverage as VDR_apo_ (black) form. Moreover, CTA-018 (green) displays a smaller region, particularly along the PC1 plane than the remaining compounds. These results demonstrate that VDR_CTA-018_ (green) illustrates a lower degree of flexibility than the remaining models as a consequence of the most stable hydrogen bond interaction.

#### Root mean square fluctuation (RMSF)

**Fig 5:**
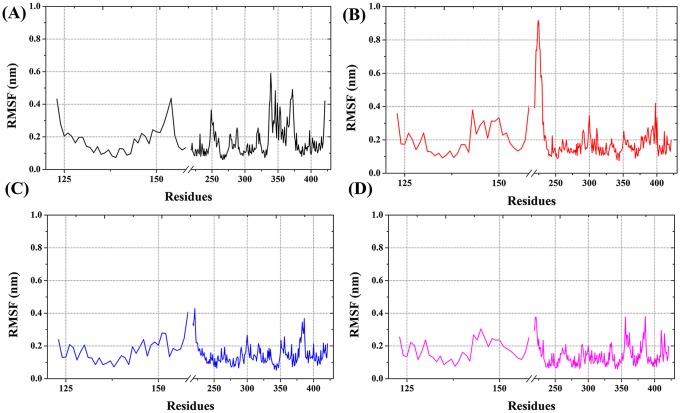
The root mean square fluctuation of VDR models VDR_apo_ (black), VDR_1α25(OH)2D3_ (red), VDR_CTA-091_ (blue), VDR_CTA-018_(pink).

In the RMSF plot we focused the positions His301 and His393. In the VDR complex, vigorous fluctuation is observed at the 210–225^th^ positions. Further, the complex RMSF fluctuates (Fig 5b, 5c and 5d) more than the apo form (except 325–375 regions) (Fig 5a). At the His301 and His393 positions, the ligand bound form fluctuated more than the apo form. The hydrogen bond interaction between—OH group and His301, His393 leads to greater fluctuation. However, in Ser233, the bound form was less flexible than the apo form of the protein. A strong hydrogen bond interaction with either His301 or His393 led to this change. Especially in the region His301, the CTA-091 bound form produced stable fluctuation (0.15 nm) as in apo form (0.10nm). In the His393 position, we could not find significant changes in both the structures (0.13 nm and 0.13 nm). In the Ser233 position, the VDR_CTA-091_complex fluctuated more (0.16 nm) than the apo form (0.08 nm). In the VDR_CTA-018_ complex, we focused mainly on the His393 region. The CTA-018 bound complex (0.10 nm) produced less fluctuation than the apo form (0.13 nm). Overall, the results indicated that the newly identified compounds were less flexible than the VDR-1α,25(OH)_2_D_3_ complex form. The RMSF of all the VDR models is shown in Fig 5.

#### Ligand binding site

The expression of VDR mutation (His301Phe, His393Phe) clearly exhibits the role of these amino acids in agonistic and antagonistic activity. The dynamic behavior of the His301and His393 in VDR was analyzed by calculating the distance between the compounds and N, O and H atoms of the key residues at the binding site for all the 200 ns simulations.

**Fig 6:**
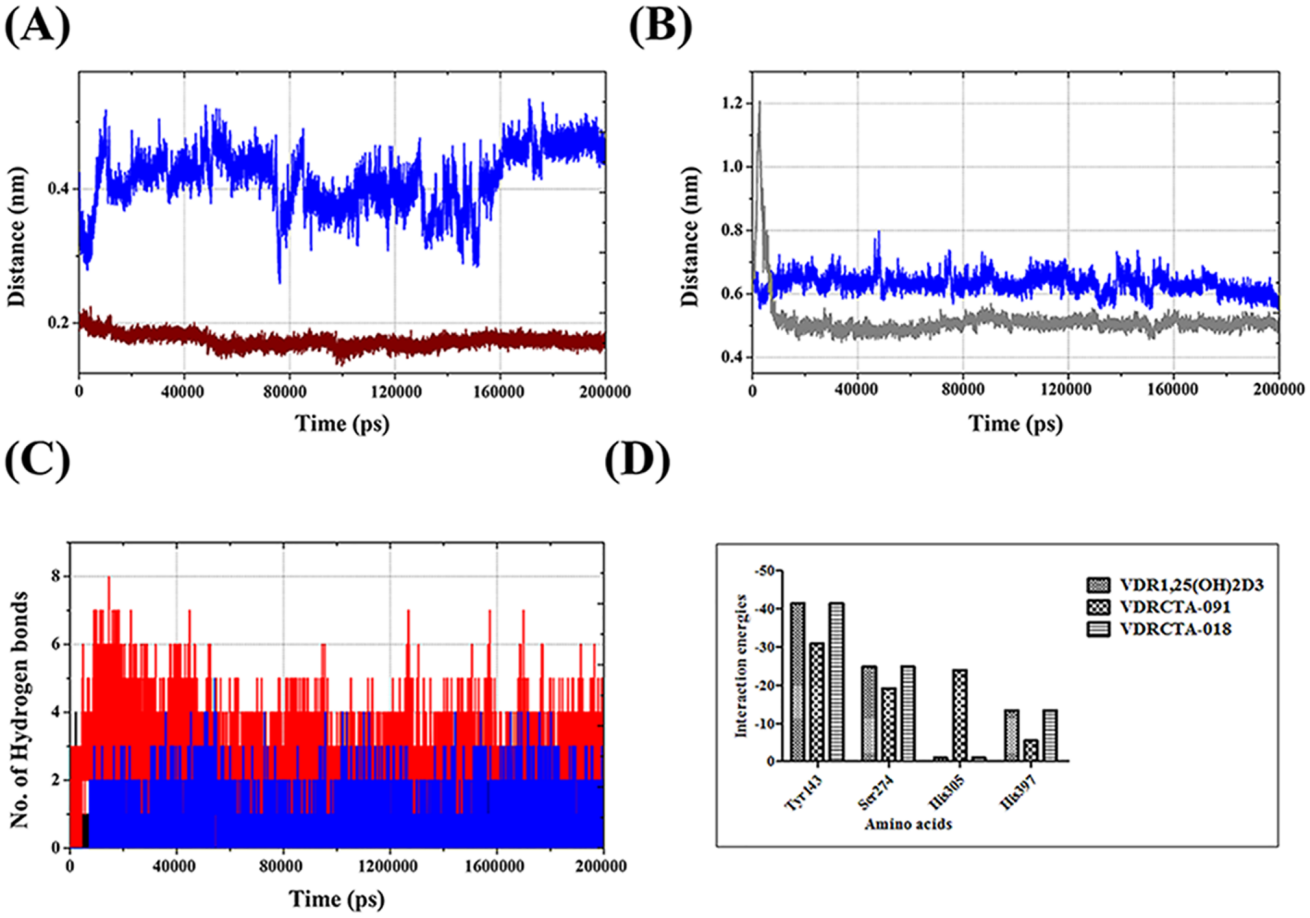
Trajectory plot of the distance between (A) 1α25(OH)_2_D_3_:(H)^**…**^N:His301 of VDR_1α25(OH)2D3_ (blue) and VDR_CTA-091_:(H)^**…**^N:His301 (wine) (B) VDR_1α25(OH)2D3_:(H)^**…**^O:His393 (blue) and VDR_CTA-018_:(O)^**…**^H:His393 (gray); (C) The total number of hydrogen bonds in VDR binding site VDR_1α25(OH)2D3_ (black), VDR_CTA-091_ (red), VDR_CTA-018_(blue). (D) Calculated hydrogen bond Interaction energies for VDR models.

The distances between the relevant heteroatoms of His301 and His393 in VDR were analyzed for molecular docking. The final MD structures involved in binding of the compounds are collectively shown in Table 3. A significant contraction and expansion were observed in MD simulation studies. These data indicate the importance of the His301 and His393 amino acids for VDR activity. The temporal changes between the selected interatomic distances were calculated. The obtained results are shown graphically in Fig 6.

**Table 3:** Molecular docking and molecular dynamics (MD) interatomic distances (nm) at the binding site of VDR.

**VDR**						
**Atom pairs**	**1,α25(OH)**_**2**_**D**_**3**_		**CTA-091**		**CTA-018**	
	**Docking (nm)**	MD* (nm)	**Docking** **(nm)**	MD* (nm)	**Docking** **(nm)**	MD* (nm)
His301	0.194	0.42	**0.53**	**0.17**	-	-
His393	**0.194**	**0.63**	-	-	**0.21**	**0.52**
**CYP24A1**						
	**Ketaconazole**		**CTA-091**		**CTA-018**	
	**Docking** **(nm)**	MD* (nm)	**Docking** **(nm)**	MD* (nm)	**Docking** **(nm)**	MD* (nm)
Met246			0.22	0.16	0.19	0.15
Glu329	0.15	0.11	0.20	0.23		
Arg128	0.49	0.46				
Hem500	**0.42**	**1.26**	0.73	0.79	0.58	1.02

*Molecular Dynamics simulations

Significant changes were observed in both (His301 and His393) the amino acids. His393 interaction was observed in VDR_1α25(OH)2D3_ (blue) and VDR_CTA-091_ (wine) (Fig 6a). In the case of His301, we found a common interaction with VDR_1α25(OH)2D3_ (blue) and VDR_CTA-018_ (gray) (Fig 6b). The distance between the H atom and N atoms of His301 and the distance between the O and H atoms of His393 are relatively different for the three different structures over the simulation period.

Vigorous fluctuation was observed in the 1α25(OH)_2_D_3_:(H)^**…**^N:His301 (blue colored plot in Fig 6a) coordination. The RMSD fluctuated between 0.3 nm and 0.5 nm for the entire simulation period. Meanwhile, the RMSD fluctuated at 0.6 nm for the entire simulation period in the context of VDR_1α25(OH)2D3_:(H)^**…**^O:His393 (blue colored plot in Fig 6b). This change is mainly due to the presence of hydrogen bond acceptor and hydrogen bond donor at the same functional group (-OH). In the compound CTA-091, we could find a potential effect on VDR_CTA-091_:(H)^**…**^N:His301 (wine color in Fig 6a). The RMSD was stabilized around 0.2 nm for the entire simulation period. Therefore, CTA-091 may act as antagonist with the constant support of His301 interaction. The presence of the hydrogen bond donor in the side chain (-NH) gives constant support for the formation of hydrogen bond interaction with His301. In the case of CTA-018, initially, the RMSD fluctuated up to 1.2 nm (gray color in Fig 6b), but after a small interval it maintained stable distance. The average settling was at ~0.30nm. Thus, the CTA-018 compound exhibits better agonistic activity in relation to 1α25(OH)_2_D_3_ and CTA-091.

#### Hydrogen bonding at the catalytic site

The 200 ns MD simulation trajectories were analyzed to gain information about the nature of the potential H-bonding interactions at the ligand binding site. The total number of hydrogen bonds was calculated for all the VDR and CYP24A1 complexes with default cutoff of 3.5 Å. An average of 4 H-bonds (maximum 8 and minimum 1) was found in three different VDR models. Of these, VDR_1α25(OH)2D3_ and VDR_CTA-091_ formed the maximum number of H-bonds over VDR_CTA-018_. As 1α25(OH)_2_D_3_ and CTA-091 possess one hydrogen bond acceptor and one hydrogen bond donor, while the CTA-018 compound possesses two hydrogen bond acceptors, the compounds 1α25(OH)_2_D_3_ and CTA-091 produced the maximum hydrogen bonds over CTA-018. The presence of one hydrogen bond acceptor and one hydrogen bond donor formed stable conformation with His301 and His393. But, in the case of CTA-018, two oxygen atoms from the O = S = O group alternatively formed hydrogen bond interaction only with His301 and not with His393 (Fig 6c).

#### Interaction energies

Hydrogen bond interactions play a major role in different chemical and biological processes, especially in ligand binding and enzyme catalysis. These interactions also influence the binding specificity, ADME properties of small molecules. Hydrogen bond interactions are the most stable in biological macromolecules because of their flexible and ubiquitous nature. Glide incorporates the contribution of Coulomb and vdW interaction energies between receptor and ligand. The binding affinity may also depend on the type and quality of the hydrogen bond formed and net electrostatic interaction energies (Even though these are considered as small and typically are neglected in scoring functions, possibly include long range effects). In this study, the hydrogen bond interaction energies were calculated in the appropriate binding pocket of protein-ligand complexes. The hydrogen bond interaction energies are shown in Table 4 and Fig 6d. In the VDR models, the contribution of His393 and His301 were focused; like His301with CTA-091 yielded more energy than 1α25 (OH)_2_D_3_ (blue) and CTA-018; further, His393 1α25(OH)_2_D_3_ and CTA-018 compounds yielded more energy than CTA-091.

**Table 4:** Interaction energies between functionally important residues and the drug molecules in VDR active site.

**VDR**			
**Interactions**	**1α25(OH)**_**2**_**D**_**3**_	**CTA-091**	**CTA-018**
Tyr143*	-41.40	**-30.90**	**-41.40**
Ser274*	-24.85	**-19.13**	**-24.85**
His301*	**-0.93**	**-23.91**	-0.93
His393*	**-13.35**	5.44	**-13.35**
**CYP24A1**			
**Interactions**	**Ketaconazole**	**CTA-091**	**CTA-018**
Met246**	-26.22	**-1.32**	**-22.17**
Glu329**	**8.45**	**-544.67**	-52.93
Arg128^†^	**23.84**	375.244	-64.10
Hem500^†^	**-46.52**	-668.79	-5.47

(Based on the bond energy, the formed hydrogen bonds are classified into *Strong interactions (4–15 Kcal/mol); **Very strong interactions (15–40 Kcal/mol); ^†^Weak interactions (<4 Kcal/mol). Source: [43]).

#### Secondary structure analysis

**Fig 7:**
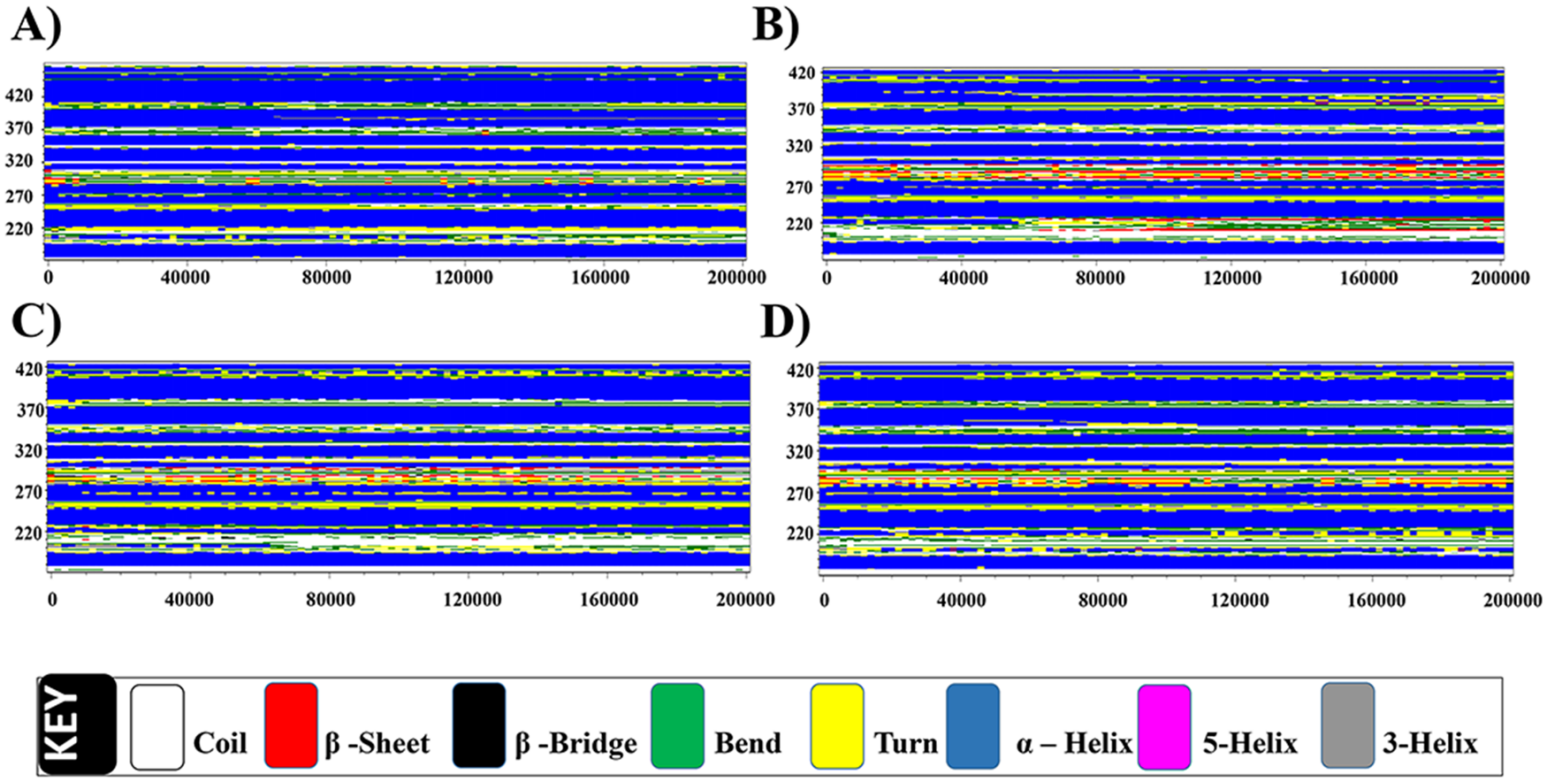
Time evolution of secondary structure analysis of (A) VDR_apo_ (B) VDR_1α25(OH)2D3_(C) VDR_CTA-091_ and (D) VDR_CTA-018_.

The temporal change in the secondary structure elements (α-helix and β-sheets) of the VDR model was analyzed with GROMACS. The results are shown graphically in Fig 7. The α-helical (blue) conformation is conserved throughout the simulation period. All the ligand bound models possessed bend to β-sheet change in between 160–240. All the ligand bound models possessed similar type of secondary structure prediction (7b—1α25(OH)_2_D_3_; 7c –CTA-091; 7d –CTA-018) in comparison with apo form (Fig 7a).

#### Binding free energy analysis

The MM-GBSA was calculated from the results of energetic analyses of 100 equal frames from the trajectory taken from each of the three MD simulations. The results of the MM-GBSA are shown in Table 5. The calculated free energies (ΔG_bind_) of the VDR/1α25(OH)_2_D_3,_ VDR/CTA-091, and VDR/CTA-018 are -63.24, -98.21, and -99.12 kcal/mol respectively. According to the energy components of the binding free energies, van der Waals are the major favorable contributors to ligand binding and nonpolar solvation terms (ΔGsolv_SA_), whereas polar solvation (ΔGsolv_GB_) opposes binding. In all the complexes, the average ΔGsolv_SA_ was very strong with the value of > 50kcal/mol, which clearly shows that ΔGsolv_SA_ is the driving force for ligand binding. As described previously, the presence of the sulfate group (O = S = O) plays an important role in the activity of the CTA-018.

**Table 5:** The MM-GBSA binding free energies (kcal/mol) of three different VDR and CYP24A1 models.

Contribution	VDR_1α25(OH)2D3_		VDR_CTA-091_		VDR_CTA-018_		CYP24A1 _ketoconazle_		CYP24A1 _CTA-091_		CYP24A1 _CTA-018_	
	Mean	STD	Mean	STD	Mean	STD	Mean	STD	Mean	STD	Mean	STD
ΔG_bind_^a^	-63.24	3.45	-98.21	3.28	-99.12	4.21	-95.65	5.22	-90.45	5.52	-92.69	6.15
ΔG_vdW_^b^	-21.42	5.11	-52.74	48.32	-21.98	8.72	-48.98	5.01	-60.89	5.11	-52.61	3.86
ΔG_solvGB_^c^	10.98	2.98	12.11	4.65	21.30	4.16	38.52	5.62	12.18	4.68	21.54	5.89
ΔG_Coulomb_^d^	-8.75	5.11	26.85	6.61	-4.85	5.85	-6.25	4.55	-6.44	5.96	1.08	6.45
ΔG_Covalent_^e^	4.11	4.32	20.88	4.38	2.16	2.86	28.63	42.93	26.85	19.26	16.80	48.77
ΔG_solLipo(SA)_^f^	-59.21	8.32	-105.1	6.33	8.21	8.88	-102.65	8.86	-82.65	5.65	-85.86	6.21

^a^Free energy of binding.

^b^Free energy of binding from the van der waals energy.

^c^Contribution to the free energy of binding from the generalized born electrostatic solvation energy.

^d^Contribution to the free energy of binding from the Coulomb energy.

^e^Contribution to the free energy of binding from the covalent energy (internal energy).

^f^Contribution to the free energy of binding from the surface area due to lipophilic energy (nonpolar contribution estimated by solvent accessible surface area).

The average ΔGsolv_SA_ was very strong with the value of > 50kcal/mol, which clearly shows that ΔGsolv_SA_ is the driving force for ligand binding.

### Molecular docking and molecular dynamics-Phase II

In the Phase-II, we displayed and discussed the results for the CYP24A1 models.

#### Molecular docking and binding mode analysis of the CYP24A1 active site

**Fig 8:**
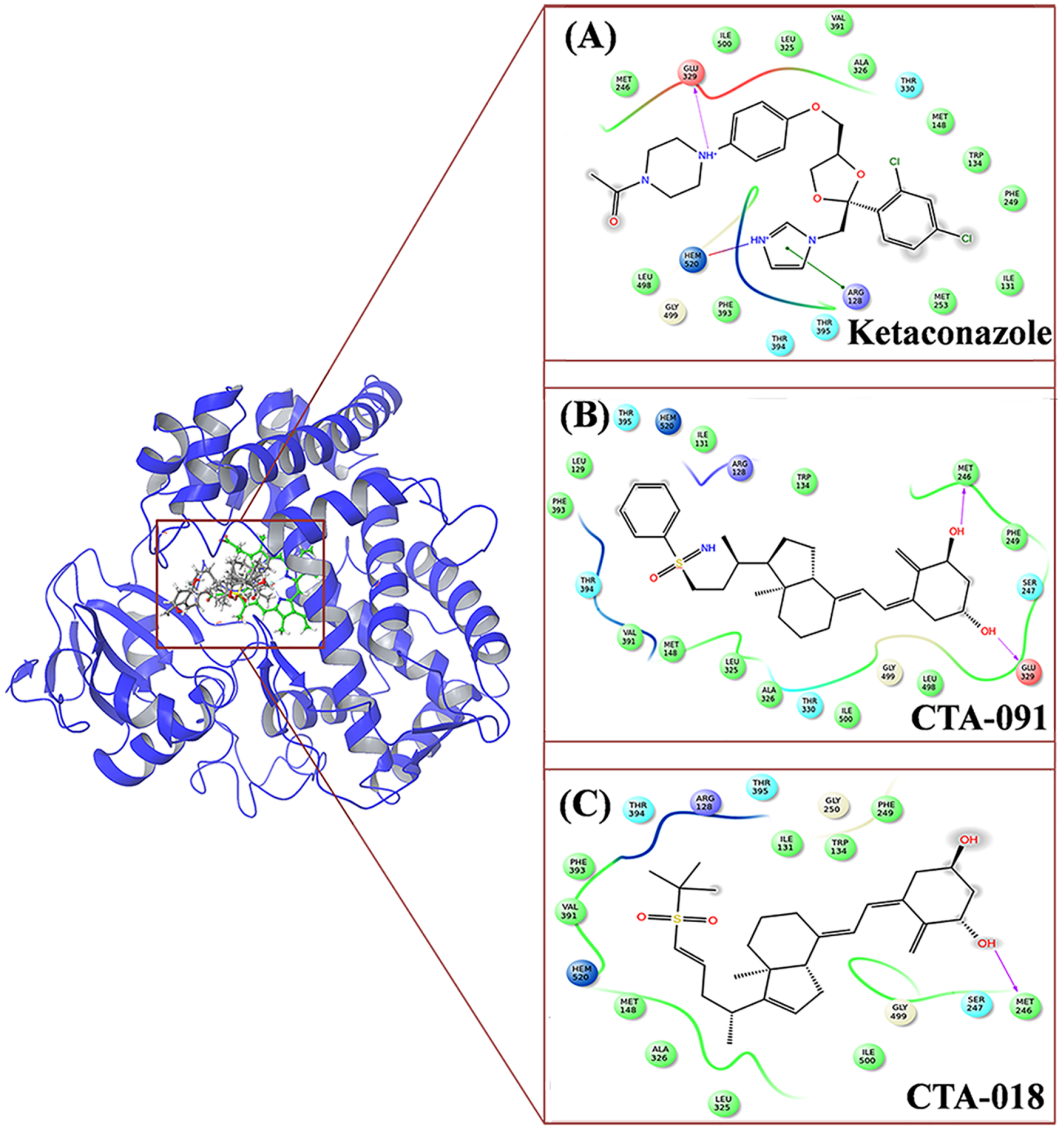
The 2D representation of the compounds (A) Ketaconazole, (B) CTA-091 and (C) CTA-018 in the active site of CYP24A1.

In the second set, ketoconazole, CTA-091, and CTA-018 were docked in the active site of the CYP24A1 (PDB id– 3K9V) [23] protein. In the case of CYP24A1, the binding mode of the newly identified vitamin D analogues was entirely different from the native compound (ketoconazole). In CYP24A1_ketoconozole_ (Fig 8a)_,_ negatively charged Glu329 (0.16 nm, C = O^**…**^NH) has a hydrogen bond interaction with ketoconazole. Another π-π stack pairing was observed between ligand and positively charged with Arg128. A salt bridge was also observed between HEM500 with the ketoconazole. In CYP24A1_CTA-091_ (Fig 8b), two different interactions were found. The carboxy group of charged sulfur containing Met246 interacted with a hydroxyl group (-OH) in the ligand molecule (0.22 nm, C = O^**…**^ HO). Another interaction was observed between negatively charged Glu329 with the CTA091 (0.21 nm, C = O^**…**^HO). Finally, in CYP24A1_CTA018_ (Fig 8c)_,_ the charged Met246 was interacted with CTA-018 (0.20 nm, C = O^**…**^HO). The interaction diagram of all the drug molecules in the active site of CYP24A1 is shown in Fig 8. The docking results are shown in Table 1.

In the context of CYP24A1, the identified molecules (CTA-091 and CTA-018) shared different binding patterns compared to ketoconazole. Further, molecular dynamics simulation studies were performed to study the importance of the three different ligands in the active site of CYP24A1.

#### Blind docking analysis

In case of CYP24A1, also we performed blind docking without importing active site information. We used induced fit docking protocol to dock all three molecules viz., ketaconazole, CTA-091 and CTA-018 in CYP24A1 protein and it binds the active site of the protein and confirms CYP24A1 antagonistic activity. The binding mode of the three different compounds in the active site of CYP24A1 is depicted in the S4 Fig.

#### Structural flexibility in CYP24A1 protein

**Fig 9:**
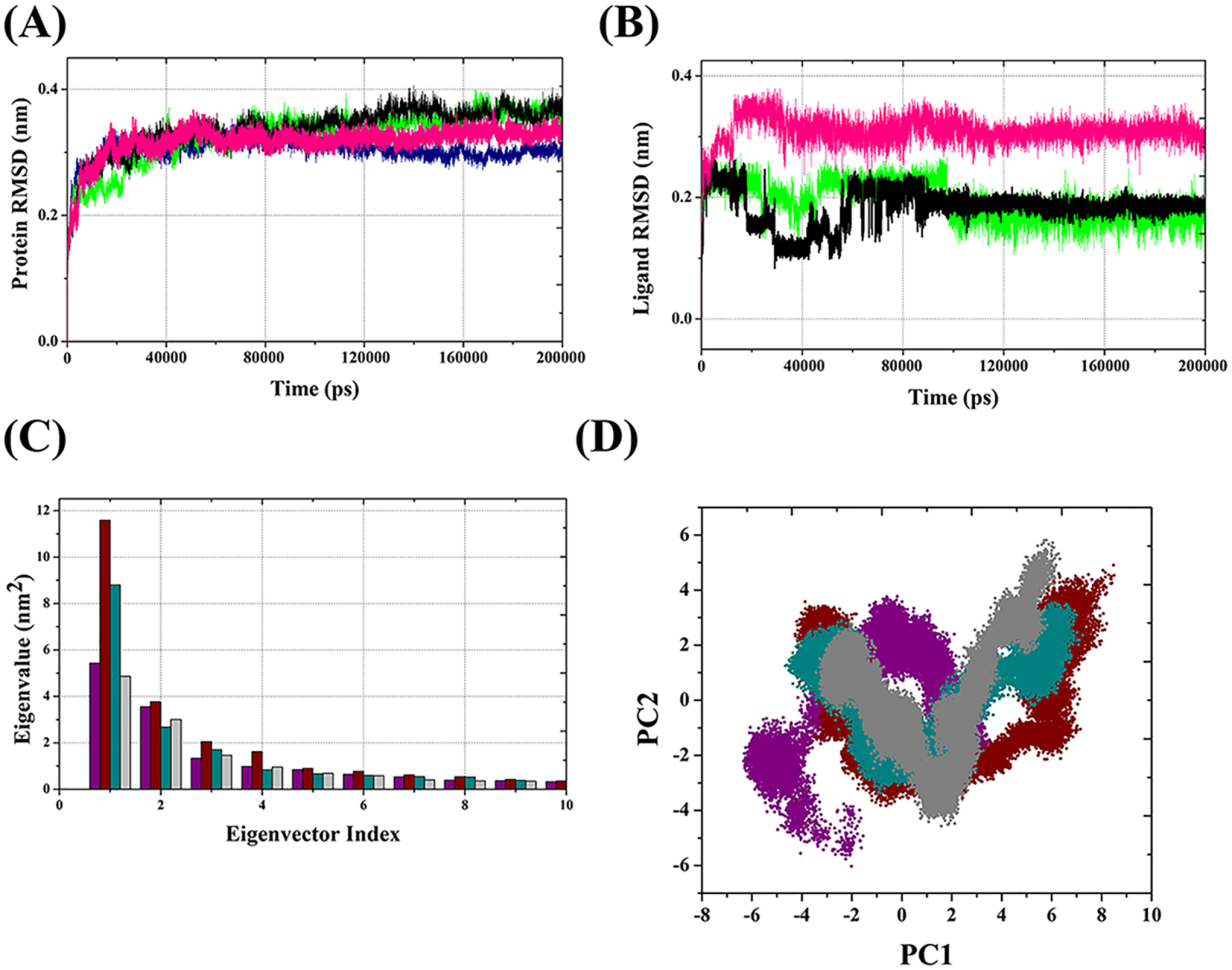
Time evolution of backbone RMSD (A) and ligand RMSD (B) of CYP24A1 models. CYP24A1_apo_ (blue), CYP24A1_ketaconozole_ (green), CYP24A1_CTA-091_ (black), CYP24A1_CTA-018_(pink). Eigenvector index versus Eigenvalue index for the first ten eigenvectors of the VDR model (C) and Projection of the motion of CYP24A1 models (D). CYP24A1_apo_ (purple), CYP24A1_ketaconozole_ (wine), CYP24A1_CTA-091_ (dark cyan), CYP24A1_CTA-018_(gray).

In Fig 9a, the backbone RMSD plots of the three CYP24A1 models CYP24A1_apo_(blue), CYP24A1_ketoconazole_(green), CYP24A1_CTA-091_(black), and CYP24A1_CTA-018_(pink) were analyzed. The CYP24A1 _CTA-018_(pink) is relatively stable than the other two complexes (CYP24A1 _1α,25(OH)2D3_ (green), CYP24A1 _CTA-091_(black)). As described earlier, initially, in the first 5000 ps the RMSD increased up to 0.2 nm in all the three complex structures. In the CYP24A1_ketoconazole_ (green) and CYP24A1_CTA-091_ (black) complexes, the RMSD gradually increased after 5000 ps up to 0.35 nm. But in CYP24A1_CTA-018_(pink), the RMSD was relatively constant for the entire simulation time. Thus, the CTA-018 molecule produced relatively stable conformation.

#### Conformational changes of ligands in CYP24A1 active site

The dynamic behavior of the three ligand molecules (Ketoconazole (green), CTA-091 (black) and CTA-018 (pink)) was analyzed in the active site of the CYP24A1 (Fig 9b).

Based on the statistical data, a significant change was found in the CTA-018 molecule (0.30 nm) compared to ketoconazole (0.19 nm) and CTA-091 (0.18 nm). All the ligands had vigorously fluctuated up to 80 ns and after that the compounds reasonably maintained stable conformers. While, the ketoconazole compound interacted well with the HEM prosthetic group, the newly identified compounds did not. This result is strongly supported by the Posner experimental data of Posner, et al. [21]. The distance between the HEM group and the ligand is discussed later. The RMSD results of the three drug molecules in the active site of CYP24A1 protein are shown in Fig 9b. Moreover, the explicit analyses of RMSD of the ligand in 10 ns MD simulation from the initial structures are reported in Table 2 **and** S5 Fig.

#### PCA

In the context of PCA, in the CYP24A1 models, a similar type of eigenvector index as the VDR models was observed. The first two eigenvectors produced more than 90% of the collective motions of the backbone protein atoms (Fig 9c). Projections of the MD trajectories were obtained at 298K onto the principal components PC1 and PC2 of our CYP24A1 models in phase space: Fig 9d shows CYP24A1_apo_ (purple) CYP24A1_ketoconazole_ (wine), CYP24A1_CTA-091_ (dark cyan) and CYP24A1_CTA-018_(gray)_._ A comparison of the data based on the width of the point clusters shows that (a) the ligand bound form (CYP24A1_ketoconazole_ (wine), CYP24A1_CTA-091_(dark cyan), and CYP24A1_CTA-018_(gray)) produced opposite conformational space during the simulation when compared to apo from CYP24A1_apo_ (purple). This result indicates that the addition of the drug molecules stabilize the CYP24A1 protein.

#### RMSF

**Fig 10:**
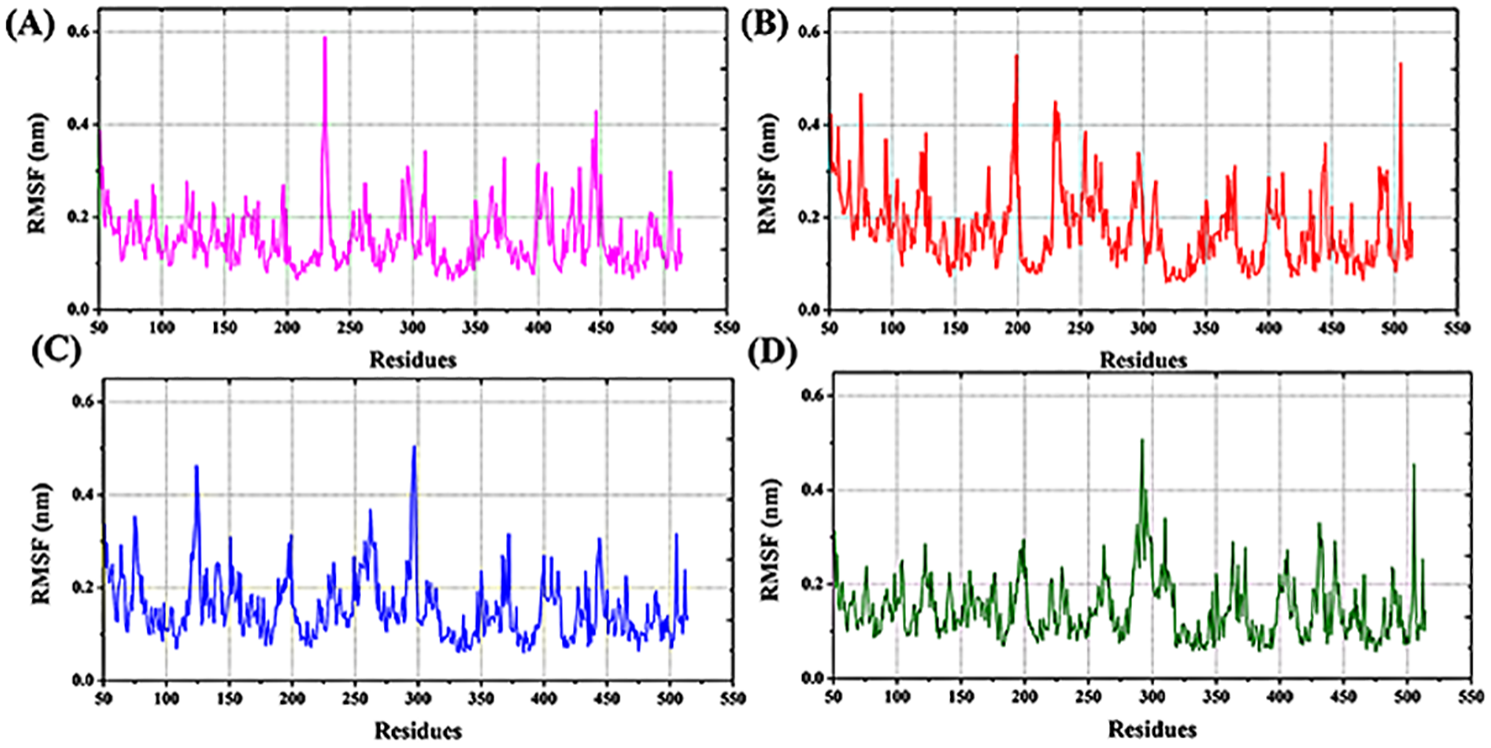
The root mean square fluctuation of CYP24A1 models CYP24A1_apo_ (gray), CYP24A1_ketoconazole_ (red), CYP24A1_CTA-091_ (blue), CYP24A1_CTA-018_(green).

The flexibility of each residue was compared by root mean square fluctuation (RMSF). The overall CYP24A1 structure contains 52% of the helical region and 9% of the beta sheet regions. A total of 24 helices and 12 strands are available in the CYP24A1 protein structure. We focused mainly on the catalytic residue changes in the protein active site. Nine regions of the CYP24A1 fold involve in the formation of the active site cavity. 13 residues in the binding cavity Ile131, Trp134, Met148, Met245, Met246, Phe249, Ala326, Glu329, Thr330, Val391, Thr394, Gly499, and Ile500 [41, 42] were identified as functionally important residues by mutational analysis. Interestingly, in this analysis, it was found that Arg128, Met246 and Glu329 interacted mainly with the compounds. In the CYP24A1_ketoconazole_ complex (red), the RMSF fluctuated more vigorously than the remaining two compounds (CTA-091 (blue) and CTA-018 (green) (Fig 10)). Vigorous fluctuation was observed between the residues 175–200 (0.45 nm) and 225–250 (0.25 nm). At the region of Met246, the CYP24A1_ketoconazole_ complex (red) (0.25 nm) was more flexible than the apo form (gray) (0.20 nm). In the CYP24A1_CTA-091_ complex, overall the CTA-091 bound complex fluctuated more compared to the apo form, especially in the region around Gly110-His140 and Gly250-Asp300. In Met246 and Glu329, the CTA-091 bound form (blue) showed relatively stable fluctuation similar to the apo form. In the CYP24A1_CTA-018_ complex (green), the RMSF was relatively stable than a few residues (Ile200-Ile500). Vigorous fluctuation was observed at the region between amino acids 225–300. Fig 10 displays the RMSF of the four different CYP24A1 models ((A) CYP24A1_apo_ (gray), (B) CYP24A1_ketoconazole_ (red), (C) CYP24A1_CTA-091 _(blue), (D) CYP24A1_CTA-018_(green).

#### Ligand binding site

**Fig 11:**
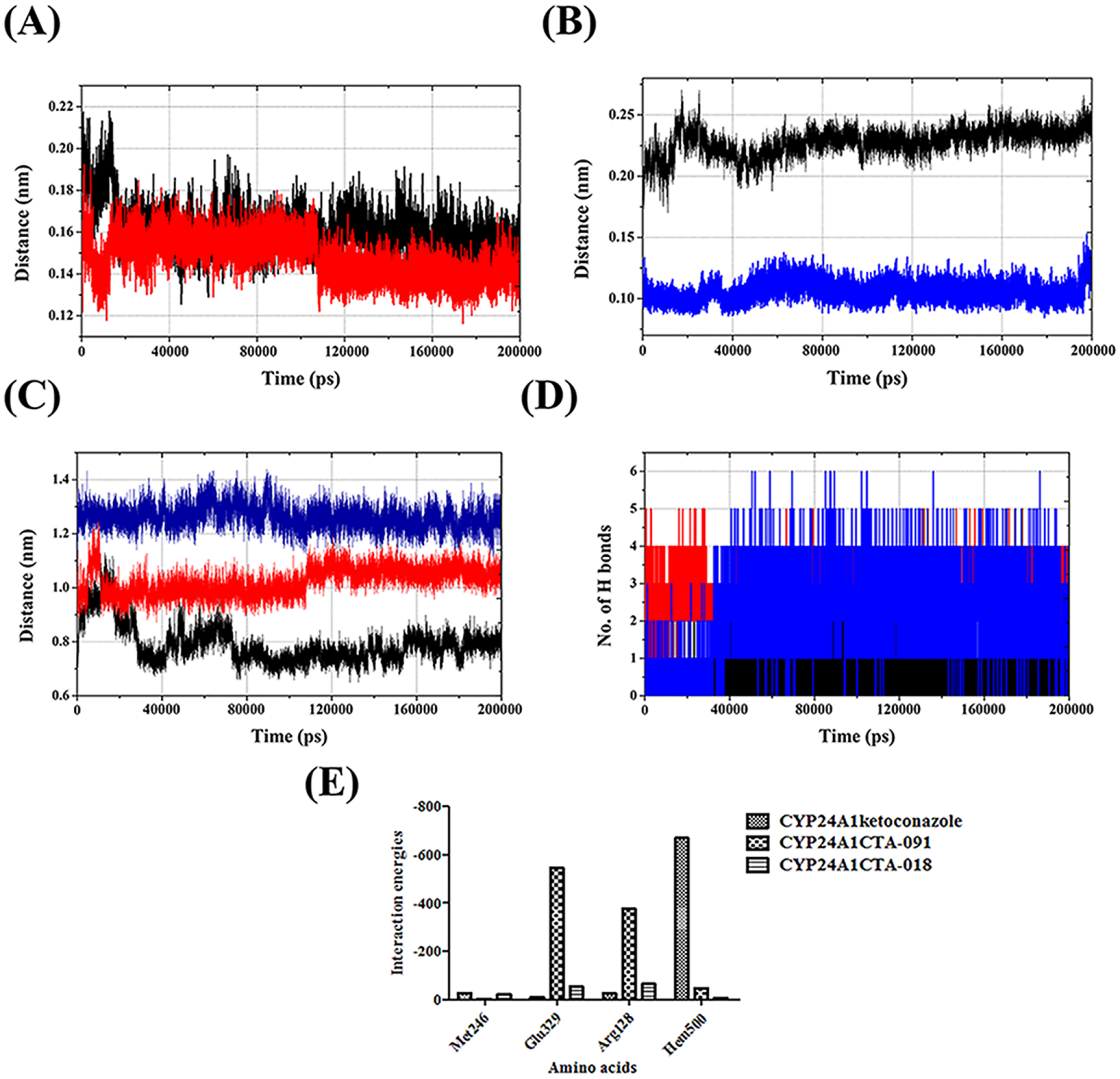
Trajectory plot of the distance between (A) CYP24A1_CTA-091_:(H)_…_(O):Met246 (black) and CYP24A1CTA-018:(H)_…_(O):Met246 (red) and (B) CYP24A1_ketoconazole_:(H)_…_(O):Glu329 (blue)(CYP24A1_CTA-091_:(O)_…_(H):Glu329(black);(C) HEM group and ketoconazole (blue), CTA-091 (black) and CTA-018 (red). (D) The total number of hydrogen bonds in CYP24A1 binding site with ketaconazole (black), CTA-091 (red) and CTA-018 (blue). (E) Calculated hydrogen bond Interaction energies for CYP24A1 models.

In the case of the CYP24A1 protein, Glu329 and Met246 amino acids were placed in the catalytic domain of the CYP24A1 site. After a rapid increase from an input distance of ~0.21 nm within the first 10ns, close CYP24A1_CTA-091_:(H)^…^(O):Met246 (black) organization was sustained at an average distance of ~0.16 nm. Similar sustainable interaction was observed in CYP24A1_CTA-018_:(H)^**…**^(O):Met246 coordination (~0.15 nm) (red). However, after 50000ps, both the distances became reasonably constant at an average of 0.16 nm and 0.15 nm respectively (Fig 11a). In the case of Glu329, CYP24A1_ketoconazole_ maintained an average distance of 0.10 nm after initial fluctuation (CYP24A1_ketoconazole_:(H)^**…**^(O):Glu329) (Blue). However, in CYP24A1_CTA-091_ the same interaction distance fluctuated vigorously over the 20000ps simulation period (CYP24A1_CTA-091_:(O)^**…**^(H):Glu329 (Black) (Fig 11b).

However, the compounds CTA-091 and CTA-018 efficiently inhibit the CYP24A1 enzyme. In order to explain this mechanism, the distance between the HEM prosthetic group and the ligand molecule (Fig 11c) were shown. The distance between heme group in HEM prosthetic group and hydrogen atom of amino group in the imidazole group CYP24A1_ketoconazole_ (blue) is ~1.2 nm–1.4nm. The CYP24A1_CTA-091_ (black) and CYP24A1_CTA-018_(red) structures maintained a distance of 0.8nm and 1.0nm and after 40000 ps it remained reasonably constant with averages of 0.9nm and 1.0 nm respectively.

This analysis clearly exhibits that the binding pattern of the vitamin D analogues CTA-091 and CTA-018 were relatively different from ketoconazole. The difference in binding pattern gives a reasonable elucidation for Posner, et al. [21] findings. CTA-091 and CTA-018 specifically target the unique substrate binding pocket of CYP24A1, unlike azole-based compounds (e.g., ketoconazole) which target the heme group at the catalytic core of the enzyme. The distances between the relevant heteroatoms of Met246, Glu329 and HEM500 in CYP24A1 were analyzed for molecular docking and the final MD structures involved in binding of the compounds are collectively reported in Table 3.

#### Hydrogen bonding at the catalytic site

The number of hydrogen bonds was analyzed between the three drug molecules with the CYP24A1 protein. The newly identified compounds produced more hydrogen bonds than ketoconazole. The ketoconazole compound (black) produced 1–3 hydrogen bonds, while CTA-091 (red) (minimum 1 and maximum 5) and CTA-018 (blue) (minimum 1 and maximum 6) produced more hydrogen bonds for the entire simulation period (Fig 11d).

#### Interaction energies

In CYP24A1 models, the newly identified compounds (CTA-091 and CTA-018) efficiently suppress the CYP24A1 enzyme. Glu329 and Met246 amino acids play a vital role in the active site of CYP24A1 enzyme. Calculation of the interaction energies between these amino acids and CYP24A1 enzyme show that in Met246, ketoconazole and CTA-018 yielded the best interaction energies. In Glu329 and Arg128, CTA-091 and CTA-018 (Gray) produced more energy. Moreover, the interaction between the HEM group and ligand molecule was high in ketaconazole when compared to CTA-091 and CTA-018. As mentioned in Fig 11c, the distance is quite low in ketaconazole in comparision with CTA-091 and CTA-018. This may be the reason that the ketaconazole interaction energies are high with CYP24A1. The interaction energies of CYP24A1 models are shown in Table 4 and Fig 11e.

#### Secondary structure analysis

**Fig 12:**
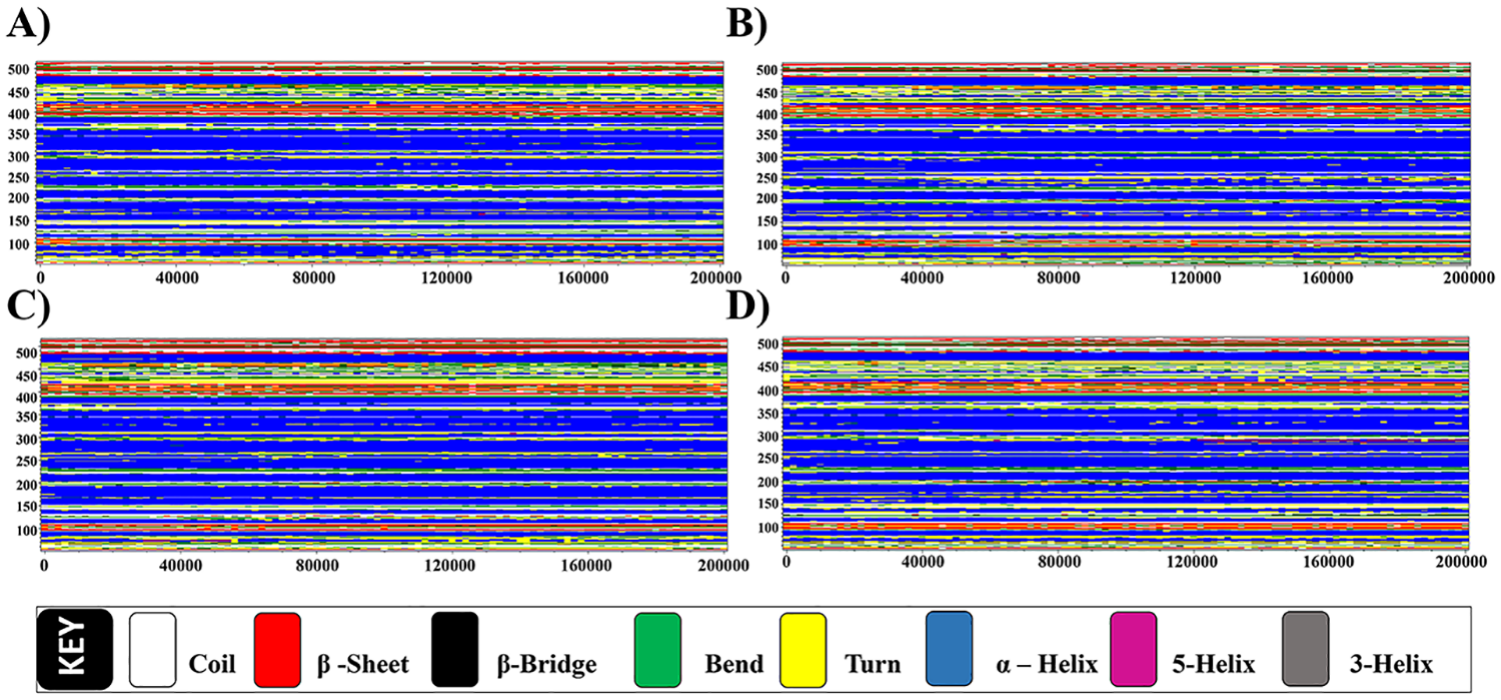
Time evolution of secondary structure analysis of (A) CYP24A1_apo_ (B) CYP24A1_ketoconazole_(C) CYP24A1_CTA-091_(D) CYP24A1_CTA-018_.

The secondary structures of the four CYP24A1 models were analyzed. The region in between the residues 150 and 400 has been fully occupied by the alpha–helix. The formation of 5-helix was observed between 160 ns and 200 ns in the CYP24A1_CTA-018_ model (Fig 12d). The residues 150–400 were stabilized after inhibitor binding attributed by the compact distribution of alpha-helix Thus, inhibitor binding accounts for low fluctuations and affecting the overall secondary structure elements. Fig 12a, 12b, 12c and 12d shows the temporal changes in the CYP24A1 models CYP24A1_apo_, CYP24A1_ketoconazole_, CYP24A1_CTA-091_ and CYP24A1_CTA-018_ respectively.

#### Binding free energy analysis

The binding free energy calculations for the three CYP24A1 complexes (CYP24A_ketoconazole_, CYP24A_CTA-091_ and CYP24A_CTA-018_) are shown in Table 5. The binding free energies for the different complexes are -95.65 kcal/mol, -90.45 kcal/mol, and -92.69 kcal/mol respectively. The CTA-018 molecule (-92.69 kcal/mol) produced good binding energy as in the native compound ketoconazole (-95.65 kcal/mol). The average ΔGsolv_SA_ was very strong and had a good value of > 50 kcal/mol. The newly identified compounds produced reasonably stable conformers and good binding affinity against the CYP24A1 protein.

#### DFT calculations

The DFT was applied to investigate the relationship between the effect of electrostatic features and the reactivity of the molecule. All the four compounds (1α25(OH)_2_D_3_, CTA-091, CTA-018, ketoconazole) were optimized at the B3LYP/6-31G**++level. Various statistical factors such as HOMO, LUMO, and MESP were calculated for all the compounds. The frontier orbitals HOMO and LUMO of chemical structures are important determinants of the compounds’ reactivity for various biological reactions.

Initially, Fukui recognized the importance of frontier orbitals as principal factors that govern the ease of chemical reactions and the stereo-chemical path, while Parr and Yang showed that most frontier theories can be rationalized from DFT.

For all the compounds, HOMO energy ranges between -0.26 eV and -0.19 eV. The high value of e-HOMO indicates the tendency of the molecule to donate the electrons in an appropriate acceptor molecule of low empty molecular orbital energy.

**Fig 13:**
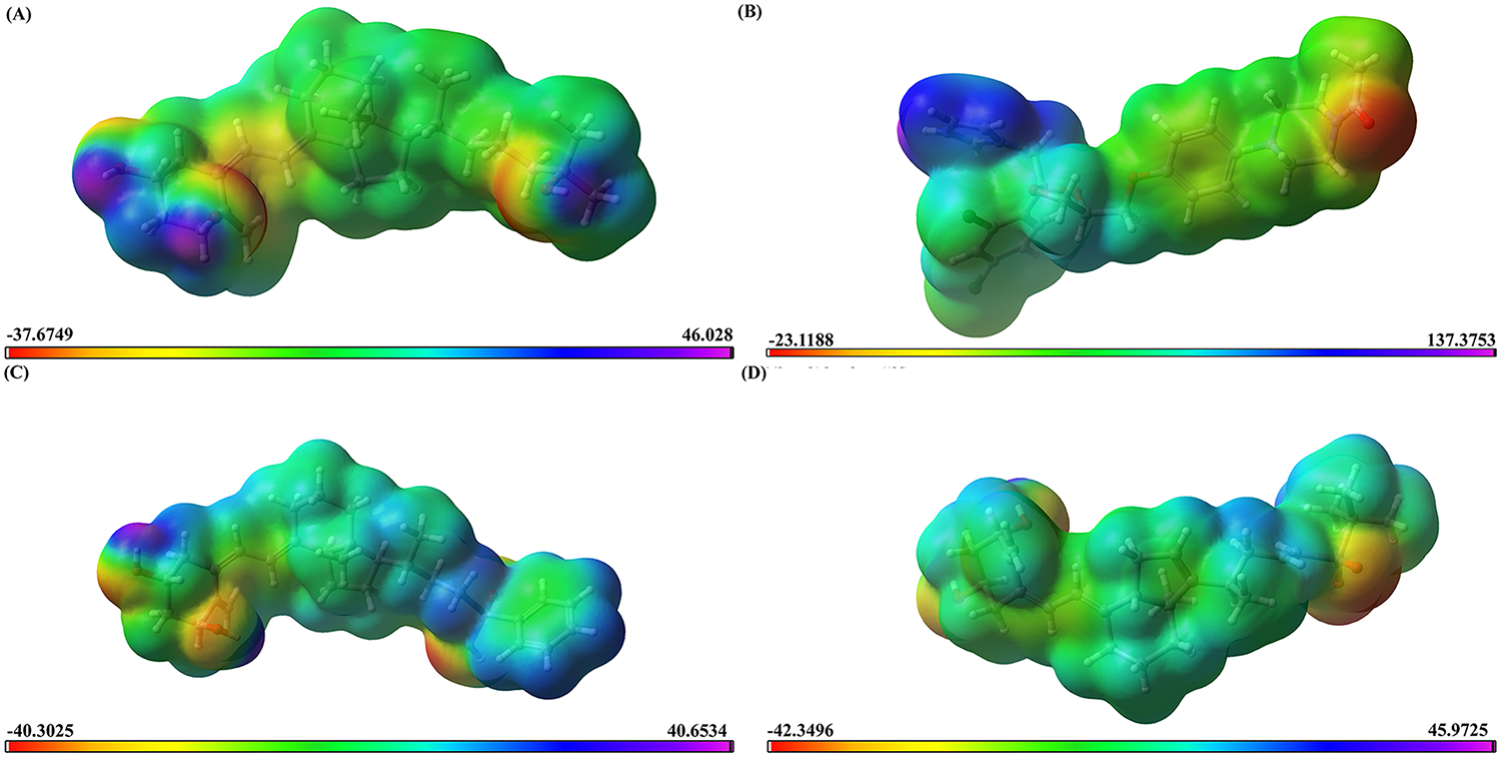
Molecular electrostatic potential superimposed onto a surface of constant electron density (0.01 e/au^3^): (A) 1,25(OH)_2_D_3_ (B) Ketoconazole (C) CTA-091 (D) CTA-018.

The HOMO-LUMO plays an important role in stabilizing the interaction between drug and receptor protein. The small HOMO LUMO gap signifies the stability of the compounds since the reactivity is high. HOMO-LUMO plots were generated to analyze the atomic contribution for these orbitals. The plots of HOMO and LUMO show the positive electron density in red color and negative electron density in blue. The entropy was relatively more in CTA-018 which showed the random rotation of this molecule and thereby the activity of the CTA-018 was significantly higher compared to the remaining compounds. The dipole moment was reasonably high in CTA-018, thus the dual compound was more reactive than the other three compounds. The HOMO values of all the compounds were more or less similar which indicated the rapid transfer of electrons. Moreover, the HOMO energy is higher when compared to the LUMO energy indicating an ability to donate the electrons rather than accept electrons with their partner receptor-binding region. Based on these results, the activity of the molecule mainly affected the thermodynamic properties such as total energy, entropy, polarity (dipole moment), and reactivity of molecules (electro negativity and LUMO energy). The DFT results are displayed in Fig 13 (Fig 13a—1,25(OH)_2_D_3_; Fig 13b—Ketoconazole; Fig 13c—CTA-091 and Fig 13d—CTA-018) and Table 6.

**Table 6:** Density Functional Theory calculations for all the druggable molecules.

Properties	Native	Ketoconazole	CTA-091	CTA-018
QM Basis	6-31G**++	6-31G**++	6-31G**++	6-31G**++
Solvation energy (kcal/mol)	-58.51	-62.31	-48.88	-60.81
HOMO (eV)	-0.20	-0.26	-0.20	-0.19
LUMO (eV)	-0.04	-0.19	-0.04	-0.03
HLG* (eV)	-0.16	-0.07	-0.16	-0.16
Zero point energy	436.32	351.21	420.32	420.31
QM dipole	2.1175	24.2078	4.3613	6.2477
Entropy (298 K)	170.22	78.58	186.21	220.11
Enthalpy (298 K)	17.24	16.85	19.65	25.21
Free energy (298 K)	-38.56	-37.85	-37.21	-46.21

* HOMO-LUMO gap

#### Dual properties of CTA-018

**Fig 14:**
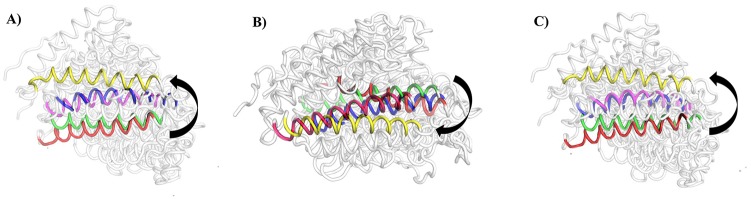
Superimposition of molecular simulated pdb structures at 40ns (red), 80ns (green), 120ns (blue), 160ns (pink), 200ns (yellow) of (A) VDR_1α25(OH)2D3_(B) VDR_CTA-091_(C) VDR_CTA-018_ respectively.

The folding helix 12 in the VDR has an important mechanism for the VDR agonistic activity. In order to prove this mechanism, we retrieved MD simulated pdb structures from VDR models in different time intervals (40ns (red), 80ns (green), 120ns (blue), 160ns (magenda), and 200ns (yellow) respectively). In Fig 14, the VDR model structures were super positioned VDR1_α25__(OH)2D3_ (Fig 14a), VDR_CTA-091_ (Fig 14b)_,_ VDR_CTA-018_ (Fig 14c) and highlighted the importance of helix 12. As explained in Fig 14, in VDR_1α25 (OH) 2D3_ and VDR_CTA-018_, the helix 12 moved close to the drug molecules that might turn into “closed” conformers. This particular change is very important for the VDR agonistic activity. However, the opposite effect was found in VDR_CTA-091_. Here, the helix 12 moved away from the active site and thereby turned into “opened” conformer. Thus, the compound CTA-091 was not able to induce the expression of VDR.

Further, in the active site of CYP24A1, the distance between the HEM group and the ligand molecule were calculated. The plot clearly explained that CTA-091 and CTA-018 had different binding pattern. This mechanism had been already explained in the previous section. The overall molecular dynamics results have given an acceptable explanation for the better activity of CTA-018.

## Conclusion

The molecular mechanism of the compound CTA-018 binding with the VDR and CYP24A1 was computationally shown and it is very well correlated with experimental studies. The conformational properties of the VDR and CYP24A1 models by performing the molecular dynamics method, principal component analysis and secondary structure analysis using Gromos9643a1 force field were observed. The results help us to understand the role of ligand groups in agonistic (VDR) and antagonistic (CYP24A1) activity.

VDR activity has been explained from the MD simulation data, which shows the binding mode of vitamin D analogues by “open” and “closed” conformations. We identified that, amino acids His301 and His393 are anxious in the agonistic and antagonistic mechanism of VDR and it is correlated with the Kakuda, et al. [22] experimental observations. Whereas CYP24A1 interaction sites of the CTA-018 molecule was differ from the previously reported drug Ketoconazole. The Compound CTA-018 shows promising dual target effect (both agonist and antagonist) mechanism compared to other compounds. The results from this study pave the way to improve our knowledge and to understand the mechanism of VDR and CYP24A1 activity at the molecular level toward decoding drugs to treat osteoporotic conditions among CKD patients.

## Supporting information

S1 Fig The binding site orientation of the 1α25(OH)_2_D_3_, CTA-091 and CTA-018 compounds along with the co-crystallized ligand TEI-9647 in the active site of VDR. (TEI-9647 –blue; 1α25(OH)_2_D3 –Orange; CTA-091 –red; CTA-018 –Purple).
(TIF)

S2 Fig Alignment of the three VDR analogues (1α25(OH)_2_D_3_, CTA-091 and CTA-018) in the active site of VDR protein. The best conformer from each complex was retrieved from the Induced Fit Docking. (Pink: 1α25(OH)_2_D_3_; Cyan: CTA-091; Orange: CTA-018).
(TIF)

S3 Fig Alignment of the four CYP24A1 inhibitors (co-crystallized ligand, ketoconazole, CTA-091 and CTA-018) in the active site of CYP24A1 protein with HEM prosthetic group. The best conformer from each complex was retrieved from the Induced Fit Docking. (Yellow: co-crystallized ligand; Green: Native; Cyan: CTA-091; Pink: CTA-018).
(TIF)

S4 Fig The average mean RMSD using bars and the corresponding standard deviations are shown using error bars for VDR models. (TIF)

S5 Fig The average mean RMSD using bars and the corresponding standard deviations are shown using error bars for CYP24A1 models. (TIF)
