# Supplementary figures and images for: A theoretical insight to understand the molecular mechanism of dual target ligand CTA-018 in the chronic kidney disease pathogenesis
Source: PLoS One. 2018 Oct 4;13(10):e0203194. doi: 10.1371/journal.pone.0203194 (PMC6171836; doi:10.1371/journal.pone.0203194)

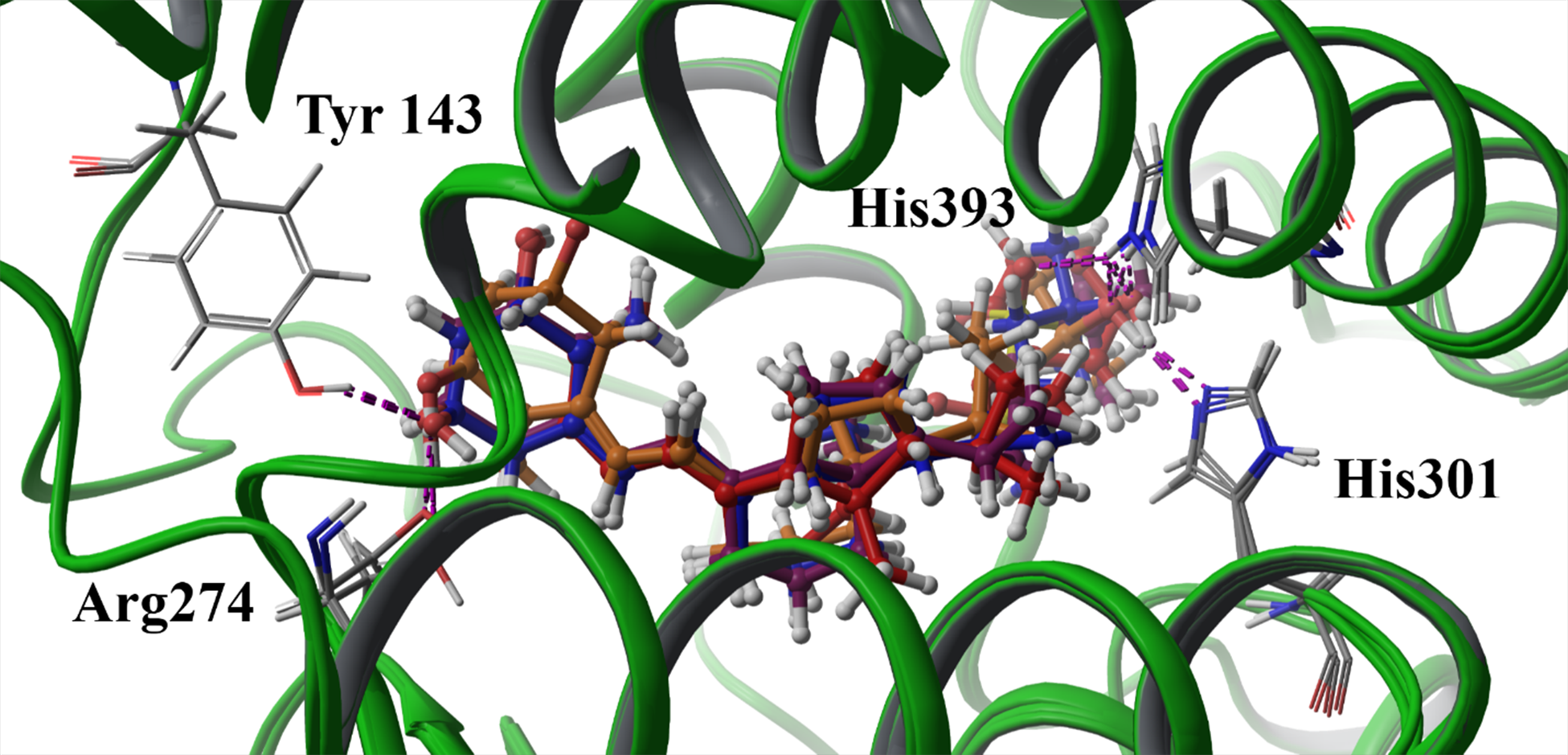

Supplement: S1 Fig — (TEI-9647 –blue; 1α25(OH)2D3 –Orange; CTA-091 –red; CTA-018 –Purple). (TIF) [file pone.0203194.s001.tif]

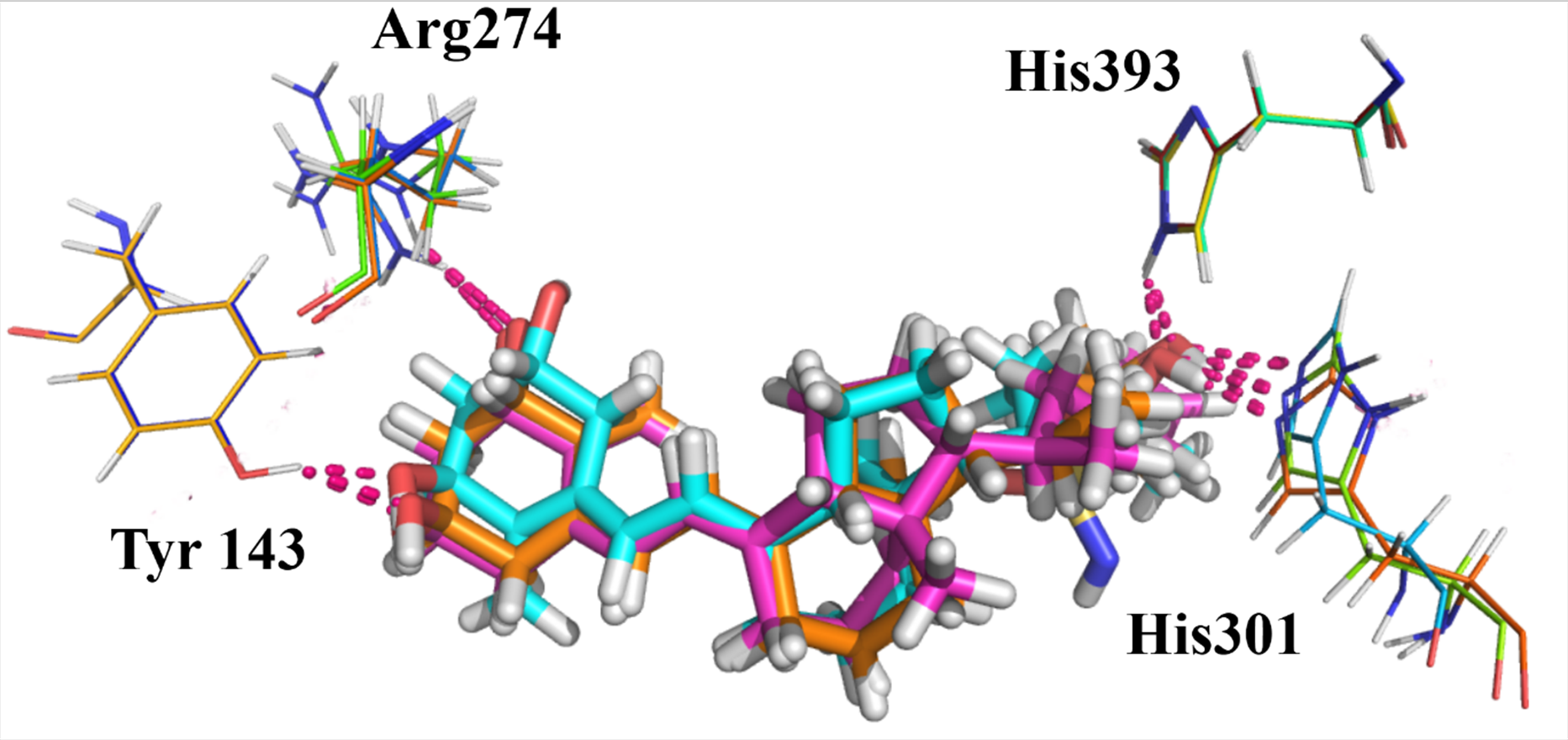

Supplement: S2 Fig — The best conformer from each complex was retrieved from the Induced Fit Docking. (Pink: 1α25(OH)2D3; Cyan: CTA-091; Orange: CTA-018). (TIF) [file pone.0203194.s002.tif]

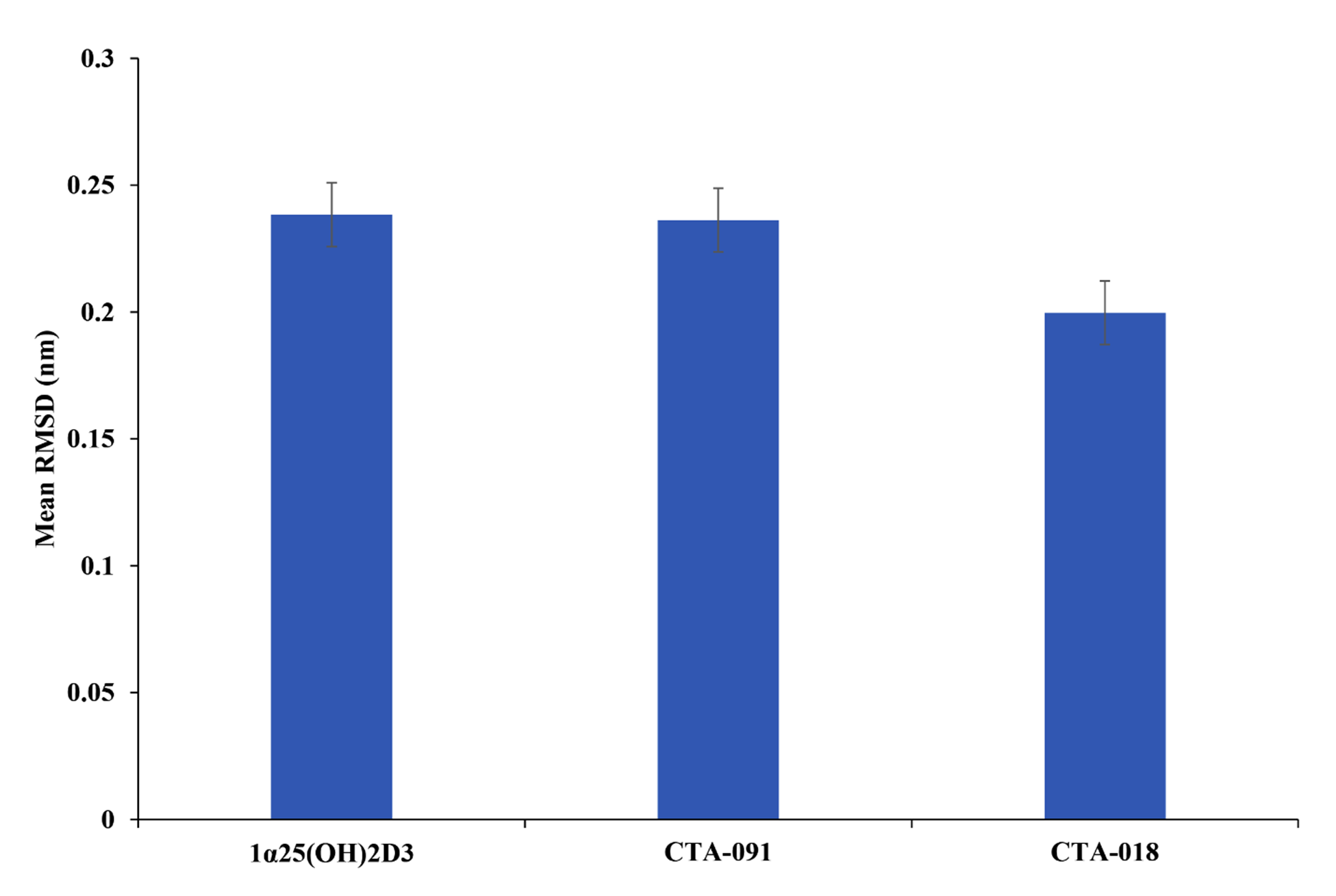

Supplement: S3 Fig — The best conformer from each complex was retrieved from the Induced Fit Docking. (Yellow: co-crystallized ligand; Green: Native; Cyan: CTA-091; Pink: CTA-018). (TIF) [file pone.0203194.s003.tif]

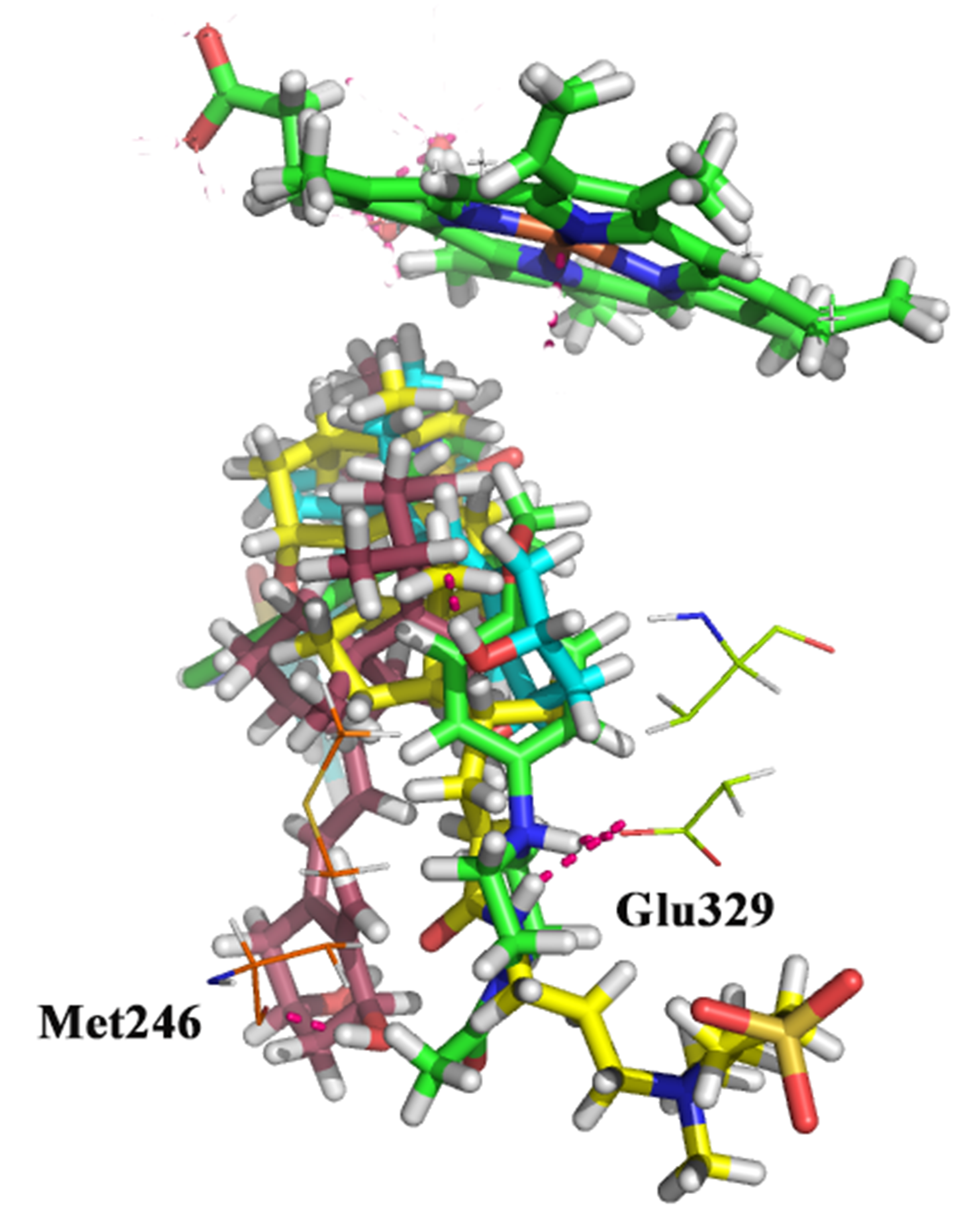

Supplement: S4 Fig — (TIF) [file pone.0203194.s004.tif]

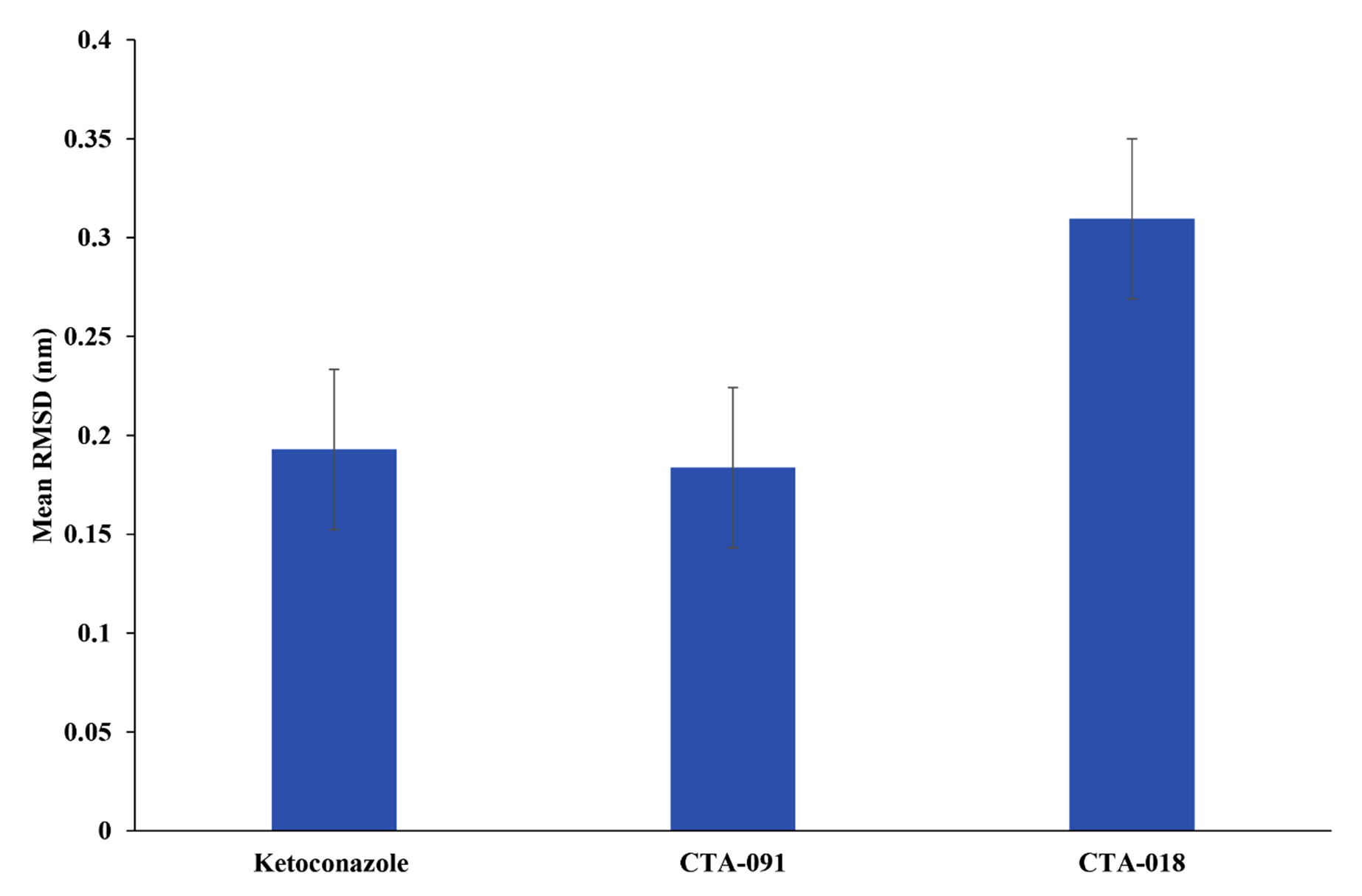

Supplement: S5 Fig — (TIF) [file pone.0203194.s005.tif]
